# Whole-genome sequencing as an investigational device for return of hereditary disease risk and pharmacogenomic results as part of the *All of Us* Research Program

**DOI:** 10.1186/s13073-022-01031-z

**Published:** 2022-03-28

**Authors:** Eric Venner, Donna Muzny, Joshua D. Smith, Kimberly Walker, Cynthia L. Neben, Christina M. Lockwood, Phillip E. Empey, Ginger A. Metcalf, Chris Kachulis, The All of Us Research Program Regulatory Working Group, Sana Mian, Anjene Musick, Heidi L. Rehm, Steven Harrison, Stacey Gabriel, Richard A. Gibbs, Deborah Nickerson, Alicia Y. Zhou, Kimberly Doheny, Bradley Ozenberger, Scott E. Topper, Niall J. Lennon

**Affiliations:** 1 Human Genome Sequencing Center, Baylor College of Medicine, Houston, TX USA; 2 Department of Genome Sciences, University of Washington, Seattle, WA USA; 3 Color Health, Burlingame, CA USA; 4 Department of Laboratory Medicine and Pathology, University of Washington, Seattle, WA USA; 5 Brotman Baty Institute for Precision Medicine, Seattle, WA USA; 6 Department of Pharmacy and Therapeutics, University of Pittsburgh School of Pharmacy, Pittsburgh, PA USA; 7 Broad Institute of MIT and Harvard, Cambridge, MA USA; 8 NIH All of Us Research Program, National Institutes of Health Office of the Director, Bethesda, MD USA; 9 Center for Genomic Medicine, Massachusetts General Hospital, Boston, MA USA; 10 Center for Inherited Disease Research, Department of Genetic Medicine, Johns Hopkins University School of Medicine, Baltimore, MD USA

## Abstract

**Background:**

The *All of Us* Research Program (AoURP, “the program”) is an initiative, sponsored by the National Institutes of Health (NIH), that aims to enroll one million people (or more) across the USA. Through repeated engagement of participants, a research resource is being created to enable a variety of future observational and interventional studies. The program has also committed to genomic data generation and returning important health-related information to participants.

**Methods:**

Whole-genome sequencing (WGS), variant calling processes, data interpretation, and return-of-results procedures had to be created and receive an Investigational Device Exemption (IDE) from the United States Food and Drug Administration (FDA). The performance of the entire workflow was assessed through the largest known cross-center, WGS-based, validation activity that was refined iteratively through interactions with the FDA over many months.

**Results:**

The accuracy and precision of the WGS process as a device for the return of certain health-related genomic results was determined to be sufficient, and an IDE was granted.

**Conclusions:**

We present here both the process of navigating the IDE application process with the FDA and the results of the validation study as a guide to future projects which may need to follow a similar path. Changes to the program in the future will be covered in supplementary submissions to the IDE and will support additional variant classes, sample types, and any expansion to the reportable regions.

**Supplementary Information:**

The online version contains supplementary material available at 10.1186/s13073-022-01031-z.

## Background

The primary objectives of the *All of Us* Research Program (AoURP, “the program”) are (1) to build a comprehensive research resource composed of surveys, biometrics, genetics, electronic health records, and biospecimens from one million or more participants reflecting the diversity of the USA; (2) to make these data and biospecimens broadly available for research exploring biological, social, and environmental determinants of health and disease; and (3) to return genomics results, gleaned from whole-genome sequencing (WGS) [1] or genotyping arrays, directly to participants who elect to receive such information. Although some previous research studies have returned genomics results to their participants [2–9] and many clinical laboratories have validated WGS pipelines for the purpose of returning germline disease diagnoses [10], none has done so on the scale, or with the diversity of participants, as the AoURP.

**Fig. 1:**
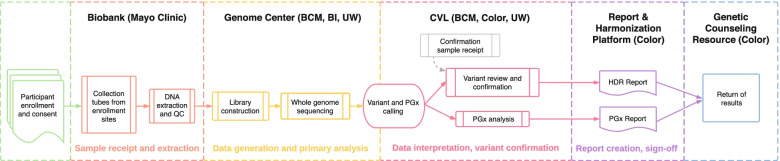
Overview of *All of Us* Research Program return of genomic results workflow. Participants are enrolled at a variety of locations including mobile sites, hospitals, and walk-in clinics. Samples are sent to the Biobank (Mayo Clinic) where DNA is extracted and stored. Upon indication from the program to proceed, samples are sent to one of three Genome Centers—Baylor College of Medicine (BCM), Broad Institute (BI), or University of Washington (UW)—for whole-genome sequencing. For participants who have consented to return of genomic results, data are forwarded on to the Clinical Validation Laboratories (CVLs) for pharmacogenomics (PGx) analysis and variant interpretation and orthogonal confirmation for Hereditary Disease Risk (HDR) gene pathogenic or likely pathogenic variants. Reports are generated by the Report and Harmonization Platform (Color) and delivered to participants through the Genetic Counseling Resource (Color). QC quality control

The program adopted the genes identified by the American College of Medical Genetics and Genomics (ACMG) for return of incidental findings [11] as the returnable regions for the “Hereditary Disease Risk (HDR) Report.” Additionally, portions of seven genes with known gene-drug interactions were chosen for return in the “Medicine and Your DNA Report” (hereafter referred to as the Pharmacogenomics or “PGx Report”). An overview of the genomics workflow for return of results is shown in Fig. 1.

During the conceptualization of the AoURP, the National Institute of Health (NIH) staff consulted with staff at the United States Food and Drug Administration (FDA), who determined that the proposed project met the criteria for a Significant Risk (SR) Device Study, defined as incorporating a device that “is for a use of substantial importance in diagnosing, curing, mitigating, or treating disease, or otherwise preventing impairment of human health and presents a potential for serious risk to the health, safety, or welfare of a subject” (21 CFR 812.3). Because of this, an Investigational Device Exemption (IDE) would be required for the return of health-related genomic results in addition to institutional review board (IRB) approval [12]. The requirement for FDA approval of NIH-sponsored research projects intending to return genetic results has been the topic of much prior discussion, primarily due to concerns over jurisdiction (i.e., FDA vs. IRB) and the non-trivial process of defining the exact requirements for approval [13]. Previous FDA approvals of direct-to-consumer genomic sequencing tests (such as Foundation Medicine [14] and 23andMe [14]) and return of results from research projects (such as NSIGHT [13]) served as useful examples, but the AoURP presented a unique set of challenges and, with its high visibility, an opportunity to establish precedents for the genomic medicine research community.

Here, we present the process of applying for and receiving an IDE from the FDA, which encompassed analysis of 1210 validation samples selected to assess the analytical validity of our whole-genome sequencing assay. These samples allowed us to assess coverage and variant calling specifically in 59 genes recommended for the return of secondary findings by the American College of Medical Genetics (ACMG). We found high reproducibility between the three genome centers involved in this analysis (differences between centers were smaller than the within center variability), with high genome-wide accuracy (99.89%) on gold standard controls. The process we defined with the FDA and the lessons learned should benefit future studies seeking to follow a similar path.

## Methods

### Interrogated regions of the genome and determination of validation requirements

The portion of the whole genome that was proposed for the return of genomic results encompassed 223,913 bases across 66 genes. Of these, 59 and 7 genes comprise the HDR and PGx reports, respectively (Additional file 1: Table S1, Additional file 2: ACMG59_PGx.combined.hg19.annotated.bed). Requirements were refined in collaboration with the FDA.

### Validation samples used

We identified 1210 DNA samples, both whole blood and buffy coat and human-derived cell lines (Table 1). These 1210 samples come from 6 different sources. The blood-derived patient samples (total *n* = 376, broad *n* = 148, BCM *n* = 189, UW *n* = 39) used at the Broad and BCM were collected as part of the eMERGE Network III study [17]. Patient samples from UW were collected by their clinical practice and originally selected due to a personal or family history of cancer. They were identified by assessing previously sequenced clinical samples from these projects for the presence of variants in the genes that will be reported in the AoURP. They are not enrolled for patient care or part of a study but do have informed consent between the provider and patient. The NGS panel originally used for these samples has been described previously [18, 19]. These samples were used to assess accuracy, precision, and assay fail rate. The blood-derived healthy donor aliquots (unique *n* = 32, broad *n* = 60, BCM *n* = 60, UW *n* = 32) were collected for the Biobank by Biochain (https://www.biochain.com/custom-services/clinical-sample-collection/) Although each genome center obtained material from 32 distinct samples, some samples were sequenced in replicate within the genome centers, as part of precision and other assessments. The Genome Centers selected matching saliva and blood samples from Biochain’s collection having the largest available quantity of DNA. These samples were used to assess the performance in the reportable range, precision, interlab concordance, limit of detection, extraction performance, molecular index performance, and assay fail rate. Genome in a Bottle (GiaB) reference cell lines [17, 20] (broad *n* = 40, BCM *n* = 34, UW *n* = 22) were ordered from the Coriell Biorepository [21]. These samples were identified by searching ground truth data for known variants in the set of genes that will be reported in the AoURP and were used to assess the performance in the reportable range, accuracy, limit of detection, molecular index performance, and assay fail rate. Cell lines with known HDR variants (broad *n* = 35, BCM *n* = 30, UW *n* = 35) were also ordered from the Coriell Biorepository [21] after having been identified by searching ground truth data for known variants in the set of genes that will be reported in the AoURP. These were used to assess the performance in the reportable range, accuracy, precision, interlab concordance, molecular index performance, and the assay fail rate. DNA from the Genetic Testing Reference Materials Coordination Program (GeT-RM) [16], which provides extensively characterized PGx cell lines (broad *n* = 155, BCM *n* = 155, UW *n* = 155). These samples were identified by searching the repository for samples containing PGx alleles that will be reported in the AoURP. They were used to evaluate the reportable range, accuracy, precision, interlab concordance molecular index performance, and assay fail rate. Lastly, we used historical, blood-derived WGS data from the TOPMed [22] project at the BCM and broad sites to evaluate sample failure rates historically. All three *All of Us* Genome Centers were previously participants in the National Heart, Lung, and Blood Institute’s Trans-Omics for Precision Medicine (TOPMed) research program and contributed WGS data for participating cohorts. TOPMed samples were sequenced at 30× minimum coverage at participating sequencing centers using harmonized methods. Because of the harmonization required in methods and QC parameters, this sample set served as an ideal resource to evaluate aggregate historical fail rates for a comparable sample type to those collected for the AoURP.

**Table 1:** Summary of assessments and numbers of specimens included in each study

Sample cohort	Assessment							
	Reportable range	Accuracy	Precision	Interlab concordance	Limit of detection	Extraction performance	Molecular index performance	Assay fail rate
Blood-derived patient samples	n/a	376	271	n/a	n/a	n/a	n/a	30
Blood-derived healthy donor aliquots	5	n/a	84	28	21	15	28	28
GIAB reference cell lines	1	18	n/a	n/a	37	n/a	37	37
Cell lines with known HDR variants	30	65	48	48	n/a	n/a	30	30
CDC GeT-RM PGx cell lines	68	310	186	186	n/a	n/a	164	175
Historical blood-derived WGS data	n/a	n/a	n/a	n/a	n/a	n/a	n/a	25,028

This table summarizes the samples that were used for each set of comparisons that comprise this study. Many samples were sequenced in replicate either across the genome centers or within them and these counts reflect replicates, not unique samples

*GIAB* Genome in a Bottle [15], *HDR* hereditary disease risk, *CDC* United States Centers for Disease Control and Prevention, *GeT-RM* Genetic Testing Reference Materials Coordination Program [16], *PGx* pharmacogenomics, *WGS* whole-genome sequencing

### Sample preparation, sequencing, and primary and secondary bioinformatics

For the control samples, we received DNA from the Coriell Biorepository [21]. DNA from commercially sourced blood samples and future AoURP participant samples was extracted from 4 ml EDTA (whole blood) or 10 ml EDTA (buffy coat) by two methods: salt-based precipitation on Autogen FlexStar or bead-based method on Chemagen 360. Samples were stored in −80 °C automated freezers and were checked for volume via a BioMicroLab volume check instrument. DNA samples were also quantified (spectrometric method) via Lunatic-Unchained Labs/Trinean DropSense 96 to obtain total DNA concentration as well as A260/280 and A260/230. All samples met a minimum concentration of 50 ng/μl and an A260/280 of 1.6–2.0. To assess the performance equivalency of the two methods, whole blood and buffy coat (WBC) specimens from five donors were extracted using the autogen and chemagen platforms. DNA from these samples was sequenced using WGS and an orthogonal targeted panel assay.

Each Genome Center performed quality control (confirmation of volume and concentration) of the samples submitted from the AoURP Biobank. Samples that met the quality thresholds were accessionned, and sample aliquots were prepared for library construction processing (normalized with respect to concentration and volume).

DNA samples were first sheared using a Covaris sonicator and then size-selected using AMPure XP beads to restrict the range of library insert sizes. Libraries were constructed using the PCR Free Kapa HyperPrep library construction kit and utilizing dual-indexed adapters. Libraries were quantified using qPCR with the Illumina Kapa DNA Quantification Kit and then normalized and pooled for sequencing. Actual implementations of the library construction processes (automation platforms used, for instance) varied across the Genome Centers. Pooled libraries were loaded on the Illumina NovaSeq 6000 instrument, and WGS was performed with Illumina reagents following the manufacturer’s best practices.

After demultiplexing, WGS analysis occurred on the DRAGEN Platform (Illumina), which consists of optimized algorithms for mapping, aligning, sorting, duplicate marking, and haplotype variant calling. Alignment used the GRCh38DH [23] reference genome, downloaded from ftp://ftp.1000genomes.ebi.ac.uk/vol1/ftp/technical/reference/GRCh38_reference_genome/. The DRAGEN pipeline produced a large number of metrics that cover lane, library, flow cell, barcode, and sample-level metrics for all runs as well as assessing contamination and mapping quality. For the purposes of the IDE analyses, the software version of the DRAGEN software was harmonized to the 3.4.12 version at all Genome Centers.

### Data analyses

#### Accuracy

In the absence of an FDA-approved ground truth assay for each reportable variant, the FDA requested that accuracy be presented as the positive and negative percent agreement (PPA and NPA, respectively) of the device calls compared to a high-quality comparator assay [24]. For comparator assays, we used clinically validated gene panels and in some cases capillary sequencing. In the case where the gene panel data was used as a comparator, calls made from the panel were designated as “true positive” and “true negative,” and calls from the WGS were categorized based on presence or absence in the panel data. Specific important variants in the population were represented in the dataset, including founder alleles in *BRCA1* and *BRCA2* (Additional file 1: Table S2).

#### Concordance of pathogenic/likely pathogenic calls in cell lines

Previously characterized human cell line-derived DNA (Coriell Biorepository) was processed through the production workflows for WGS, capillary, or panel sequencing at all Genome Centers and CVLs (BCM; UW; and Color Health, Color). Calls of the known pathogenic (P) or likely pathogenic (LP) variants were assessed at each site and the concordance of variant calls and genotypes was measured (Additional file 1: Table S3).

#### Performance in reference cell lines

Performance on the National Institute of Standards and Technology (NIST) human reference cell lines (NA12878 [15], NA24385, NA24149, and NA24143) was evaluated by comparing to the Genome in a Bottle v3.3.2 gold standard truth set. In all comparisons against reference samples, we used the vcfeval [25] tool and harmonized on the --ref-overlap --decompose and --output-mode=annotate flags. Comparisons were limited to the NIST high confidence regions. To qualify as a true positive, variants must match not only the alternate allele but also the genotype call (e.g., the zygosity). Variant calls with mismatched genotypes were considered false positives.

#### Accuracy of PGx sites

Accuracy of PGx calling was determined using 159 patient samples that had previously been orthogonally validated by Sanger sequencing, next-generation sequencing (NGS) panel, or genotyping assays (Table 3). As noted above for the HDR genes, the selected PGx clinical samples provided a representation of genes and alleles that aligned with the expected prevalence of reportable alleles in the general population as determined by Clinical Pharmacogenetics Implementation Consortium (CPIC) [26] (www.cpicpgx.org). In genes where reported allele frequencies are extremely rare and no clinical samples were accessible, cell line samples were used to provide a more comprehensive list of reportable alleles for validation (Additional file 1: Tables S4 and S5).

#### Precision

To assess the equivalence of processing and variant calling across the three Genome Centers within the AoURP device, we computed the concordance of variant calls and genotypes from five donor-derived blood specimens collected and processed through the WGS and variant calling pipelines. Replicate samples were run at each center, and the equivalence between replicates was determined to demonstrate that the variability in sample processing and variant calling between labs was no greater than the variability within labs (Additional file 1: Table S6). The overall equivalence was calculated using the Jaccard similarity coefficient between each pair of labs over all variants (i.e., the size of the intersection of the calls divided by the size of the union of the calls). We further evaluated the equivalence across and between labs using the WGS data from 175 human cell line-derived genomic DNA samples that were part of the PGx accuracy and NIST accuracy studies (Additional file 1: Table S7).

#### Inter- and intra-Genome Center precision

Two studies were completed to calculate inter- and intra-Genome Center precision. In the first study, 20 replicate blood-derived DNA samples from five individual donors were examined. Data from clinical gene panel testing [27, 28] of these samples alone, without additional confirmatory testing, was used to define the “truth.” Although this is an imperfect truth set, it allows us to directly compare to a previously validated clinical test, which we expect to be highly accurate. Variant genotypes from WGS on all 20 samples at each Genome Center were compared to the panel variants to determine concordance across sites by genomic context (Additional file 1: Table S8). In the second study, 30 human cell lines (the same cell lines as were used in the P/LP variant accuracy assessment) were sequenced utilizing the clinical NGS panel as described above to define the “truth.” Calls from WGS on all 30 samples at each Genome Center were compared to the panel variants and genotypes to determine concordance across sites by genomic context (Additional file 1: Table S9). To demonstrate the equivalence of cell line-derived DNA with that of blood-derived primary samples, we calculated performance measures and technical measures for selected assessments, run on both clinical cohorts and cell lines (Additional file 1: Tables S10 and S11).

#### Precision of PGx calling

Precision of PGx variant calling was assessed by processing 62 cell lines with known PGx alleles, as defined by Stargazer [29] at each of the three Genome Centers and CVLs (Additional file 1: Table S12).

#### Limit of detection

In order to determine the range of acceptable genomic DNA inputs into library construction, an input titration experiment using DNA derived from NA12878 with total input amounts ranging from 25 to 1500 ng into library construction was performed. These input levels span a range from 10× the lowest acceptable input to 2× the highest standard input into library construction across the Genome Centers. To assess the effect of lower input amounts on sensitivity and to confirm that the minimum input identified produces acceptable sensitivity and precision, the vcfeval tool [25] was leveraged to calculate sensitivity versus NIST for each titration point (Additional file 1: Figure S6). Similar titration and analysis were done with samples from the AoURP Biobank (Additional file 1: Table S13).

#### Sequence generation quality control specification determination

For coverage metrics, we used the Picard tool to remove read data uniformly (downsampling) from four NIST control samples for which gold standard variant data is available. For contamination, we created a set of bioinformatically contaminated samples by adding progressively more read data from a second sample to a control sample. For the duplicate rate, we progressively added more duplicates to a sample while holding the yield constant.

#### Reportable range

To define reportable genomic intervals, we selected transcripts for each of the 59 genes of interest, using primarily MANE Select [30] and RefSeq [31] guidelines. We extend exons from the − 15 upstream intronic position to the + 6 downstream intronic position. We then added intervals to cover known P/LP variants outside of the − 15 to + 6 regions and for 43 PGx star allele sites. We excluded three types of technically challenging regions from the reportable range: (1) *PMS2* exons 12–15, which has high homology with the *PMS2CL* pseudogene; (2) regions with high GC content (typically > 75% across 100 bp) that can result in low coverage; and (3) regions with spurious variant calling artifacts due to the presence of micro-repeats (di-, trinucleotides) and long homopolymers (Additional file 1: Table S14).

An additional set of regions did not consistently have at least 20× coverage in 20% of the samples in the dataset. A per-site coverage analysis was performed across the entire HDR gene and PGx site region with a dataset of 104 samples from the three Genome Centers with a whole-genome mean coverage of 30–35×. This analysis revealed six regions that included 56 bases across four genes (Additional file 1: Table S15). For these regions, none was low enough quality to consider excluding the region from the reportable range.

#### Invalid rates

To illustrate fail rates at each step, we used historical data and data from this validation study. Invalid rates were also calculated from panel data at Color and UW. BCM data represents capillary sequence data from an internal cohort. For historical data, we used the National Heart, Lung, and Blood Institute Trans-Omics for Precision Medicine (TOPMed) [22] cohort as it represents a large number of samples run at all three Genome Centers (Additional file 1: Tables S16 and S17).

### Statistics

For the precision calculation, the equivalence was calculated using the Jaccard similarity coefficient (the size of the intersection divided by the size of the union of the sample sets) between each pair of labs over all variants. Calls are considered “intersecting” if they match exactly in both the alternate allele and genotype. The union contains all variant calls in the sample sets.

#### Calculation of PPA and NPA in clinical specimens

A set of 271 patient samples was examined for accuracy across a range of genomic contexts, variant subtypes, and zygosity. Gene panels were those used in the eMERGE III study at BCM and Broad 15 or an ACMG panel at UW 16, intersected with the HDR Report interval. Genomic contexts were defined using bed files from the Genome Alliance for Genomic Health (GA4GH, www.ga4gh.org) benchmarking-tools repository. Confidence intervals were calculated using the normal distribution (i.e., mean ± 1.96 (standard deviation/square root of *n*) where *n* is the number of samples). Although this approach was accepted for this IDE by the FDA, we note that it is more correct to assume a binomial distribution. To calculate PPA and NPA, we compared each sample against a set of variant genotype truth data for that sample using vcfeval (either published variant genotypes for reference sets like NIST and Get-RM or variant genotypes from previous sequencing runs in our clinical pipelines for clinical samples). Variant genotypes appearing in the truth but not in the evaluation sample are false negatives, variant genotypes appearing in both the truth and evaluation sample are true positives, variant genotypes appearing in neither the truth nor the evaluation sample are true negatives, and variant genotypes that do not appear in the truth but appear in the evaluation sample are false positives.

## Results

### Initial assessment of FDA requirements

**Fig. 2:**
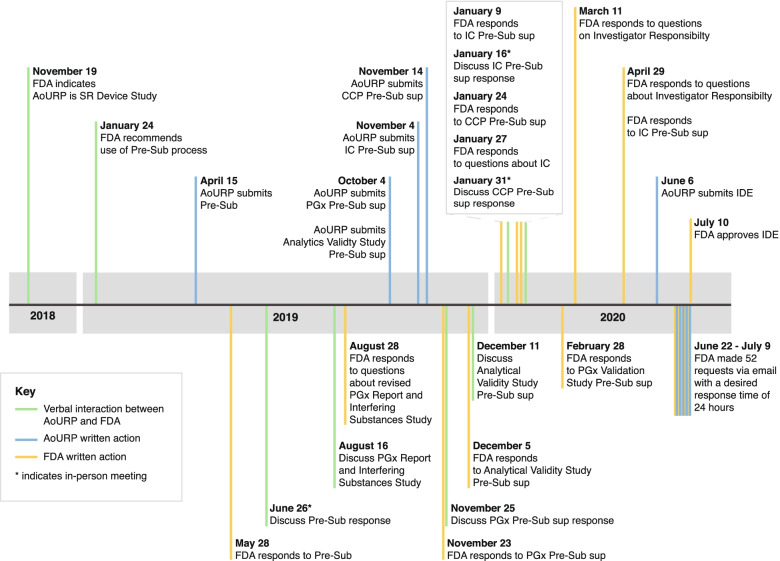
Timeline of the IDE application process. The requirements for the investigational device exemption (IDE) content were refined through a series of pre-IDE submissions and responses, in-person meetings, and teleconferences with the United States Food and Drug Administration (FDA) over a period of 19 months. AoURP, *All of Us* Research Program; CCP, Change Control Policy; IC, informed consent; PGx, pharmacogenomics; Pre-Sub, pre-submission; SR, significant risk; Sup, supplement

Over 19 months, the Genome Centers, CVLs, and NIH staff discussed each major element of the program (including participant consent, sample processing, analysis and interpretation, and return of results) with reviewers at the FDA via a series of conference calls and presubmission inquiries, to define the elements required for the IDE application (Fig. 2). The FDA determined that the “device” to be approved in this case constituted all steps of the process and requested that validation samples be primarily derived from blood to match the sample types used by the program. Those samples should reflect the variant types (single-nucleotide variants, SNVs, and insertions or deletions, indels) and genomic properties (GC rich, complexity) that were anticipated to be reported to participants. Substantial work was required even after the submission of the original IDE. During the review process, the FDA requested additional clarifications and analyses with short turn arounds (typically 3–5 days). We have paraphrased those requests in Additional file 1: Table S18 to provide an example of what investigators should expect during the review process. An analysis of the incidence of reportable variants within the HDR genes across the clinical laboratories at BCM, color, UW, and Laboratory for Molecular Medicine at Partners HealthCare (LMM, associated with the Broad Genome Center) revealed that some genes will likely have very few reportable variants and that indels are not reportable in several genes (Additional file 1: Figure S1).

Per the IDE process, requirements were set by the study sponsor (NIH), which were then used to define five specific acceptance criteria. The data showed sequencing depth exceeding 20× for the HDR and PGx reportable range, high accuracy for all Genome Centers (> 99.7%), and high concordance of data generated at the Genome Centers (> 99.7%) for both the HDR region and PGx alleles. Limit-of-detection studies demonstrated accurate data produced from a range of input amounts with an expected invalid rate for blood-derived specimens of < 1%.

### Accuracy

Across a set of 271 blood-derived clinical samples, PPA ranged from 94.24 to 100% when broken down by genomic context, with NPA at 100% across all categories (Table 2 and Additional file 1: Table S8). The accuracy of the AoURP device was > 99% (horizontal dotted line) in events up to 20 bases in length (Additional file 1: Figure S2). We detected five recurrent false-positive and false-negative variants in a large number of the samples (Additional file 1: Table S19) which were removed from the analysis. All were benign, likely benign or variants of uncertain significance, would not be reported clinically, and fall in regions that are known to have sequence homology or mapping issues [32]. On a set of human cell lines with known HDR variants, the overall concordance was 100% for a set of known P/LP variants (Additional file 1: Table S3), and in a detailed assessment of variant and genotype calling in the control sample NA12878, accuracy was 100% for variants in the reportable range (Additional file 1: Table S20) and 99.89% for variants across the whole genome (Additional file 1: Table S21). The accuracy of PGx calling was determined on 159 blood-derived patient samples which had previously been orthogonally validated in each Genome Center (Table 3). We observed concordance in 100% of calls (595/595).

**Table 2:** Accuracy of variant calling across genomic contexts and variant types in patient samples

	Panel+^*a*^/WGS–^*b*^	Panel–/WGS+	Panel+/WGS+	Panel–/WGS–	PPA [95% CI]	NPA [95% CI]
All compiled	76^*c*^	201	29,475	45,847,148	99.74% [99.68–99.81%]	100% [100–100%]
P/LP variants only	0	0	145	28,474,503	100% [100–100%]	100% [100–100%]
SNVs only	23	6	28,710	45,848,108	99.92% [99.87–99.97%]	100% [100–100%]
Insertions only	9	8	225	29,887,204	96.20% [94.30–98.00%]	100% [100–100%]
Deletions only	33	12	540	42,170,158	94.24% [92.65–95.83%]	100% [100–100%]
Segmental duplications	12	2	2119	38,466,267	99.44% [99.10–99.78%]	100% [100–100%]
Known pseudogenes	9	74	1075	45,676,596	99.17% [98.60–99.73%]	100% [100–100%]
Low mappability regions	18	12	1069	36,912,107	98.34% [97.84–98.85%]	100% [100–100%]
Low complexity regions	24	69	956	38,072,735	97.55% [96.35–98.75%]	100% [100–100%]
Low GC regions	6	12	197	14,792,575	97.04% [95.87–98.22%]	100% [100–100%]
High GC regions	0	0	23	3,891,553	100% [100–100%]	100% [100–100%]
Heterozygous variants only	75	147	18,295	45,858,382	99.59% [98.48–99.70%]	100% [100–100%]
Homozygous variants only	1	83	11,180	45,865,561	99.99% [99.97–100%]	100% [100–100%]

^*a*^*+* indicates that a variant was present on the panel or the corresponding whole genome sequencing (WGS) sample. ^*b*^– indicates that a variant was not present on the panel or the corresponding WGS sample. ^*c*^Subsequent to the investigational device exemption (IDE) submission, additional review indicated that this number should in fact be a total of 21 panel positive, but WGS negative variants distributed across the various subcategories (some variants were missannotated in the original tables). However, to reflect what was actually submitted as part of the IDE, the original number is listed here. The impact on overall performance measures is small. *PPA* positive percent agreement, *CI* confidence interval, *NPA* negative percent agreement, *P/LP* pathogenic/likely pathogenic, *SNVs* single-nucleotide variants

**Table 3:** Call concordance of PGx alleles using WGS and orthogonal methods in patient samples

Gene and star allele	Number of clinical samples	Correct calls	Incorrect calls	Correct calls	Incorrect calls	Correct calls	Incorrect calls	Correct calls	Incorrect calls	Correct calls	Incorrect calls	Correct calls	Incorrect calls	Correct calls	Incorrect calls	Overall concordance
CYP2C19*10	1	1/1	0	-	-	-	-	1/1	0	-	-	-	-	-	-	2/2
CYP2C19*17	40	25/25	0	15/15	0	-	-	25/25	0	15/15	0	-	-	-	-	80/80
CYP2C19*17/*17	1	1/1	-	-	-	-	-	1/1	-	-	-	-	-	-	-	2/2
CYP2C19*2	48	33/33	0	-	-	15/15	0	33/33	-	-	-	-	-	15/15	0	96/96
CYP2C19*2/*2	3	3/3	0	-	-	-	-	3/3	-	-	-	-	-	-	-	3/3
CYP2C19*22	1	-	-	1/1	0	-	-	-	-	1/1	0	-	-	-	-	2/2
CYP2C19*3	3	3/3	0	-	-	-	-	3/3	-	-	-	-	-	-	-	3/3
CYP2C19*35	1	1/1	0	-	-	-	-	1/1	-	-	-	-	-	-	-	1/1
CYP2C19*4	6	3/3	0	3/3	0	-	-	3/3	0	3/3	0	-	-	-	-	12/12
CYP2C19*8	1	-	-	-	-	1/1	0	-	-	-	-	-	-	1/1	0	2/2
CYP2C19*9	2	-	-	2/2	0	-	-	-	-	2/2	0	-	-	-	-	4/4
*DPYD c.1129-5923C>G*	2	2/2	0					2/2	-	-	-	-	-	-	-	4/4
DPYD c.1679T>G (*13)	3	3/3	0					3/3	-	-	-	-	-	-	-	6/6
*DPYD c.2846A>T*	2	-	-	2/2	0	-	-	-	-	2/2	0	-	-	-	-	4/4
DPYD*2 (c.1905+1G>A)	2	-	-	2/2	0	-	-	-	-	2/2	0	-	-	-	-	4/4
*G6PD A-202A_376G*	3	3/3	0	-	-	-	-	3/3	0	-	-	-	-	-	-	6/6
*G6PD* Canton, Taiwan-Hakka, Gifu-like, Agrigento-like	1	1/1	0	-	-	-	-	1/1	0	-	-	-	-	-	-	2/2
*G6PD* Ilesha	2	2/2	0	-	-	-	-	2/2	0	-	-	-	-	-	-	4/4
*G6PD* Mediterranean, Dallas, Panama, Sassari, Cagliari, Birmingham	5	5/5	0	-	-	-	-	5/5	0	-	-	-	-	-	-	10/10
*G6PD* Seattle, Lodi, Modena, Ferrara II, Athens-like	1	1/1	0	-	-	-	-	1/1	0	-	-	-	-	-	-	2/2
*G6PD* Union, Maewo, Chinese-2, Kalo	2	2/2	0	-	-	-	-	2/2	0	-	-	-	-	-	-	4/4
NUDT15*2	2	2/2	0	-	-	-	-	2/2	0	-	-	-	-	-	-	4/4
NUDT15*3	9	8/8	0	-	-	1/1	0	8/8	0	-	-	-	-	1/1	0	18/18
SLCO1B1*15	28	22/22	0	-	-	6/6	0	22/22	0	-	-	-	-	6/6	0	56/56
SLCO1B1*15/*15	4	4/4	0	-	-			4/4	0	-	-	-	-	-	-	8/8
SLCO1B1*17	3	-	-	-	-	3/3	0	-	-	-	-	-	-	3/3	0	6/6
SLCO1B1*5	8	7/7	0	-	-	1/1	0	7/7	0	-	-	-	-	1/1	0	16/16
TPMT*2	6	5/5	0	1/1	0	-	-	5/5	0	1/1	0	-	-	-	-	12/12
TPMT*3A	9	9/9	0	-	-	-	-	9/9	0	-	-	-	-	-	-	18/18
TPMT*3C	5	5/5	0	-	-	-	-	5/5	0	-	-	-	-	-	-	10/10
UGT1A1*27	3	3/3	0	-	-	-	-	3/3	0	-	-	-	-	-	-	6/6
UGT1A1*28	70	37/37	0	33/33	0	-	-	37/37	0	33/33	0	-	-	-	-	140/140
UGT1A1*36	12	4/4	0	8/8	0	-	-	4/4	0	8/8	0	-	-	-	-	24/24
UGT1A1*36/*36	1	1/1	0	-	-	-	-	1/1	0	-	-	-	-	-	-	2/2
UGT1A1*37	3	2/2	0	-	-	1/1	0	2/2	0	-	-	1/1	0	-	-	6/6
UGT1A1*6	8	3/3	0	5/5	0	-	-	3/3	0	5/5	0	-	-	-	-	16/16
		**Broad**^*a*^ **(WGS**^*b*^**)**		**BCM**^*c*^ **(WGS)**		**UW**^*d*^ **(WGS)**		**Color**^*e*^ **CVL**^*f*^ **(Panel)**		**BCM CVL (Sanger)**		**UW CVL (Panel)**		**UW CVL (Array)**		

^*a*^*BI* Broad Institute, ^*b*^*WGS* whole-genome sequencing, ^*c*^*BCM* Baylor College of Medicine, ^*d*^*UW* University of Washington, ^*e*^*Color* Color Health, ^*f*^*CVL* Clinical Validation Laboratory

### Precision

The Genome Centers jointly agreed to harmonize the production pipelines to the extent possible, but some parts of the pipelines (e.g., DNA quality control [QC] and quantification) could not be harmonized perfectly, so we used sample replicates to demonstrate functional equivalence. Twenty commercially obtained, blood-derived healthy donor aliquots from five individuals were examined, with clinical, gene-panel testing of these samples used to define “truth.” WGS variants and genotypes were compared to the panel variants to determine concordance across sites and discordance by genomic context (Additional file 1: Table S8). The data were highly concordant, with the differences observed between Genome Centers smaller than the within-center sample variance. When broken out by genomic context, the context with the largest range was insertions, where PPA varied between 94.44 and 100%.

PGx variant calling precision across the three Genome Centers was assessed by processing 62 cell lines with known PGx alleles. A total of 298 of 300 alleles were found to be concordant, with discordance only found in *G6PD* alleles due to an incorrect ploidy call in the bioinformatics pipeline (Additional file 1: Table S12).

### Limit of detection

The minimum amount of input DNA for sequencing was determined from NA12878 to be 250 ng, as lower amounts either failed to produce a library or showed reduced sensitivity. Additionally, four commercially obtained, healthy donor aliquots from the AoURP Biobank were compared against that sample’s gene panel data, with assessments of sensitivity and precision (Additional file 1: Table S13). We observed a range of PPA from 98.1% for indels in DNA samples isolated on the Autogen FlexStar instrument to 100% for indels in DNA samples isolated on Chemagen 360 instruments. NPA remained at 100% across these comparisons.

To evaluate the performance as a function of allele fraction, seven replicates of NA12878 were sequenced with the AoURP WGS pipeline and compared to results from NIST, restricted to the high confidence regions. The analysis indicates that confident and accurate heterozygous calls are made between 30 and 75% allele fraction for SNVs and 30–65% allele fraction for indels (Additional file 1: Figure S8).

#### Other analyses

We performed several additional analyses to satisfy the FDA requirements. We assessed equivalent performance and technical measures of cell line-derived DNA with that of blood-derived DNA. Clinical specimens and cell lines were highly concordant (Additional file 1: Tables S10 and S11). We demonstrated that the intended analyte was being measured by noting that the K_2_EDTA blood collection kits used by the program were approved by the FDA for hematological blood analysis. We described how quality-metric thresholds were selected and optimized (Figs. S3 and S4), showed that variant performance from both extraction methods and both blood collections were equivalent (Additional file 1: Table S22), and determined a fail rate below 1% for blood-derived specimens. Finally, we established the accuracy of the “liftover” step (conversion of variants called on the GRCh38DH reference to the GRCh37 reference), with the exception of four sites that had no corresponding GRCh37 position (Additional file 1: Table S23).

#### Change management

In concordance with FDA regulations [33], the AoURP established a Change Control Policy, which stated that each proposed change will be assessed for risk by an expert review panel made up of members from the AoURP Genome Centers and CVLs, and a formal recommendation is submitted for NIH evaluation and approval prior to FDA involvement. Categories are summarized in Additional file 1: Table S24. For major changes (e.g., new reportable genes, changing procedure QC metrics and acceptance criteria, addition of new concepts to reports), the AoURP will obtain FDA approval through a supplemental application to the parent IDE. “Moderate” changes, defined as those that do not affect the validity of the data (see 21 CFR 812.35(a)(3)(ii) for complete definition) require FDA notification within 5 working days of implementation. Minor changes (defined fully in 21 CFR 812.150(b)(5)) may be reported to the FDA in the annual report.

## Discussion

The FDA IDE process for the return of genomic results required nearly 2 years to complete but ultimately demonstrated that WGS as a clinical laboratory assay performed at a high level across all three Genome Centers, genes of interest, and various sequence contexts. Based on previous FDA submissions from Foundation Medicine [34], MSK-IMPACT [35], and 23andMe [36], as well as the experience in the NSIGHT project [13] and the FDA’s guidelines [24], we initially expected the requirements for the IDE process to be largely similar to the Clinical Laboratory Improvement Amendments (CLIA) validation framework under which the AoURP Genome Centers and CVLs operate their clinical laboratories (Additional file 1: Table S25). Instead, we found that the FDA requirements exceed those that are required by CLIA.

Genomic testing research studies under the supervision of IRB protocols, as the AoURP is, have historically not been subject to IDE regulation [37]. However, in 2013, the FDA asserted authority over the use of genomic sequence of newborns with the NSIGHT project [13]. AoU leadership consulted the FDA early on because of the unique scale of return of results activities in the AoURP. The FDA determined that since the safety and effectiveness of these investigational devices for the purposes of providing information on a person’s health has not been established (i.e., it is unknown whether these devices are safe and effective for the claimed use), returning results directly to study subjects in which they or their healthcare providers may make medical decisions based upon the results, present significant risks to the study subjects, and thus would require FDA oversight. An IDE was preferable to full approval because the device is not commercially marketed and will likely undergo significant modifications during its course. Our experience has shown that any group initiating a research project that may require FDA approval should budget considerable upfront time and resources to the process. This is particularly true if the “device” has no predicate in FDA approval history. The complexity of defining, designing, and executing on a validation study of this scale ultimately required that we reduce the proposed scope in several important ways (e.g., limiting acceptable specimen input types to blood, returning only SNVs and Indels, reducing reportable PGx alleles).

A major impediment to efficient approval was the lack of a closely related predicate test (i.e., a test that has previously been reviewed and approved by the FDA). Previously reviewed NGS and genotyping assays (e.g., Foundation Medicine, 23andMe) were different enough as to not be considered predicates. The FDA staff extrapolated from tests and technologies that they had previously reviewed, such as genotyping arrays and targeted sequencing.

Professional organizations provide guidelines for clinical laboratories who are validating genomic assays [38, 39]. The FDA’s approach differs from these guidelines in important ways. First, in defining “ground truth,” the FDA strongly prefers patient-derived clinical specimens over reference samples derived from cell lines. In this study, the FDA requested a comparison to demonstrate the equivalence of these two sample types (Tables S10 and S11). That comparison showed near equivalence; however, it is unclear if this will impact the FDA’s preference for patient-derived samples going forward. Second, assessing variant calling performance for a particular variant class in a representative number of sites is generally considered acceptable as a proxy for the performance of that variant class at other genomic positions [38, 39]. However, the FDA required us to demonstrate performance in every reportable gene and PGx allele. The three Genome Centers collectively had access to hundreds of clinical remnant specimens from previous studies; for a smaller group or a single laboratory applying for an IDE, this would have been a challenging requirement.

This work represents the first time a sequencing consortium has harmonized the bioinformatics pipelines at the level of the software version and command-line parameters instead of focusing on “functional equivalence.” This allowed us to capitalize on one another’s validations and greatly simplified concordance calculations. However, one potential downside is the lack of “multiple views” of the same data set, provided by multiple independent analysis approaches, which can support one another where they agree and potentially reveal systematic problems where they do not.

## Conclusions

The AoURP is a groundbreaking research project that will generate a massive dataset to accelerate the study of disease but which also presents challenges under the current regulatory landscape. After a multi-year effort across multiple groups, the AoURP device was validated and an IDE was granted so that the genomics arm of the program could begin. We appreciate the collaborative relationship with the FDA and hope that this work will provide a streamlined model for future projects.

## Supplementary Information

**Additional file 1.** Supplementary Tables S1-S25 and Supplementary Figures S1-S8.

**Additional file 2.** Reportable region in bed format.

## Data Availability

eMERGE III patient samples are accessible via dbGap(). Other patient samples are not shareable due to cell lines and GeT-RM samples can be ordered from the Coriell biorepository. eMERGE III ground truth data are available in dbGAP under under phs001616.v1.p1. eMERGE III WGS data, and UW clinical data are not available due to patient confidentiality. The vcfeval program is available from https://github.com/RealTimeGenomics/rtg-tools^25^. GA4GH benchmarking tools, including bed files, are available from https://github.com/ga4gh/benchmarking-tools [40]. NIST reference data is available from https://ftp-trace.ncbi.nlm.nih.gov/giab/ftp/release/. GeT-RM [16] samples can be ordered from the coriell institute: http://www.coriell.org/1/NIGMS/Additional-Resources/Multiply-Confirmed-Mutations-GeT-RM [41]. Reference genome data is available from ftp://ftp.1000genomes.ebi.ac.uk/vol1/ftp/technical/reference/GRCh38_reference_genome/ [42].

## References

[1] The precision medicine initiative cohort program – building a research foundation for 21st century medicine. https://www.nih.gov/sites/default/files/research-training/initiatives/pmi/pmi-working-group-report-20150917-2.pdf. Accessed 13 May 2021.

[2] Carere DA (2017). Prescription medication changes following direct-to-consumer personal genomic testing: findings from the Impact of Personal Genomics (PGen) Study. Genet Med.

[3] Carey DJ (2016). The Geisinger MyCode community health initiative: an electronic health record-linked biobank for precision medicine research. Genet Med.

[4] eMERGE Clinical Annotation Working Group (2020). Frequency of genomic secondary findings among 21,915 eMERGE network participants. Genet Med.

[5] Hart MR (2019). Secondary findings from clinical genomic sequencing: prevalence, patient perspectives, family history assessment, and health-care costs from a multisite study. Genet Med.

[6] Roberts JS (2018). Patient understanding of, satisfaction with, and perceived utility of whole-genome sequencing: findings from the MedSeq Project. Genet Med.

[7] Sanderson SC (2017). Psychological and behavioural impact of returning personal results from whole-genome sequencing: the HealthSeq project. Eur J Hum Genet.

[8] Vassy JL (2017). The impact of whole-genome sequencing on the primary care and outcomes of healthy adult patients: a pilot randomized trial. Ann Intern Med.

[9] Zoltick ES (2019). Predispositional genome sequencing in healthy adults: design, participant characteristics, and early outcomes of the PeopleSeq Consortium. Genome Med.

[10] Marshall CR (2020). Best practices for the analytical validation of clinical whole-genome sequencing intended for the diagnosis of germline disease. NPJ Genom Med.

[11] Kalia SS (2016). Recommendations for reporting of secondary findings in clinical exome and genome sequencing, 2016 update (ACMG SF v2.0): a policy statement of the American College of Medical Genetics and Genomics. Genet Med.

[12] Information Sheet Guidance For IRBs, Clinical Investigators, and Sponsors. http://www.fda.gov/downloads/RegulatoryInformation/Guidances/UCM126418.pdf. Accessed 13 May, 2021.

[13] Milko LV (2019). FDA oversight of NSIGHT genomic research: the need for an integrated systems approach to regulation. NPJ Genom Med.

[14] EVALUATION OF AUTOMATIC CLASS III DESIGNATION FOR The 23andMe Personal Genome Service (PGS) Genetic Health Risk Test for Hereditary Thrombophilia, Alpha-1 Antitrypsin Deficiency, Alzheimer’s Disease, Parkinson’s Disease, Gaucher Disease Type 1, Factor XI Deficiency, Celiac Disease, G6PD Deficiency, Hereditary Hemochromatosis and Early-Onset Primary Dystonia. https://www.accessdata.fda.gov/cdrh_docs/reviews/den160026.pdf. Accessed 13 May 2021.

[15] Zook JM (2019). An open resource for accurately benchmarking small variant and reference calls. Nat Biotechnol.

[16] Pratt VM (2016). Characterization of 137 genomic DNA reference materials for 28 pharmacogenetic genes: a GeT-RM collaborative project. J Mol Diagn.

[17] Zouk H, et al. Harmonizing clinical sequencing and interpretation for the eMERGE III Network. Am J Hum Genet. 2019;105(3):588–605. 10.1016/j.ajhg.2019.07.018 PMC6731372 31447099

[18] Pritchard CC (2012). ColoSeq provides comprehensive lynch and polyposis syndrome mutational analysis using massively parallel sequencing. J Mol Diagn.

[19] Pritchard CC (2014). Validation and implementation of targeted capture and sequencing for the detection of actionable mutation, copy number variation, and gene rearrangement in clinical cancer specimens. J Mol Diagn.

[20] Zook JM (2014). Integrating human sequence data sets provides a resource of benchmark SNP and indel genotype calls. Nat Biotechnol.

[21] Coriell Institute. www.coriell.org. Accessed 31 Jan 2022.

[22] Taliun D (2021). Sequencing of 53,831 diverse genomes from the NHLBI TOPMed Program. Nature.

[23] Lowy-Gallego E (2019). Variant calling on the GRCh38 assembly with the data from phase three of the 1000 Genomes Project. Wellcome Open Res.

[24] Center for Devices & Radiological Health (2020). Considerations for design, development, and analytical validation of N.

[25] Cleary JG, et al. Comparing variant call files for performance benchmarking of next-generation sequencing variant calling pipelines. bioRxiv. 2015;023754. 10.1101/023754.

[26] Relling MV (2020). The clinical pharmacogenetics implementation consortium: 10 years later. Clin Pharmacol Ther.

[27] Neben CL, et al. Multi-gene panel testing of 23,179 individuals for hereditary cancer risk identifies pathogenic variant carriers missed by current genetic testing guidelines. J Mol Diagn. 2019;21(4):646–57. 10.1016/j.jmoldx.2019.03.001 31201024

[28] Berger MJ, et al. Color Data v2: a user-friendly, open-access database with hereditary cancer and hereditary cardiovascular conditions datasets. Cold Spring Harbor Laboratory. 2020 2020.01.15.907212. 10.1101/2020.01.15.907212. 10.1093/database/baaa083 PMC7661094 33181822

[29] Lee S-B, Wheeler MM, Thummel KE, Nickerson DA (2019). Calling star alleles with stargazer in 28 pharmacogenes with whole genome sequences. Clin Pharmacol Ther.

[30] Matched annotation from NCBI and EMBL-EBI (MANE). https://www.ncbi.nlm.nih.gov/refseq/MANE/. Accessed 13 May 2021.

[31] Pruitt KD, Tatusova T, Maglott DR (2007). NCBI reference sequences (RefSeq): a curated non-redundant sequence database of genomes, transcripts and proteins. Nucleic Acids Res.

[32] Mandelker D (2016). Navigating highly homologous genes in a molecular diagnostic setting: a resource for clinical next-generation sequencing. Genet Med.

[33] CFR - Code of Federal Regulations Title 21. [cited 2021 Mar 8]; Available from: https://www.accessdata.fda.gov/scripts/cdrh/cfdocs/cfcfr/CFRSearch.cfm?fr=812.35

[34] FoundationOne CDx. https://www.accessdata.fda.gov/scripts/cdrh/cfdocs/cfpma/pma.cfm?id=p170019. Accessed 13 May 2021.

[35] Next generation sequencing based tumor profiling test. https://www.accessdata.fda.gov/scripts/cdrh/cfdocs/cfpmn/denovo.cfm?ID=DEN170058. Accessed 13 May 2021.

[36] 23andMe Personal Genome Service (PGS) Pharmacogenetic Reports. https://www.accessdata.fda.gov/cdrh_docs/pdf19/K193492.pdf. Accessed 13 May 2021.

[37] Evans BJ (2015). The limits of FDA’s authority to regulate clinical research involving high-throughput DNA sequencing. Food Drug Law J.

[38] Gargis AS (2012). Assuring the quality of next-generation sequencing in clinical laboratory practice. Nat Biotechnol.

[39] Rehm HL (2013). ACMG clinical laboratory standards for next-generation sequencing. Genet Med.

[40] ga4gh. GitHub - ga4gh/benchmarking-tools: Repository for the GA4GH Benchmarking Team work developing standardized benchmarking methods for germline small variant calls. *GitHub* https://github.com/ga4gh/benchmarking-tools. Accessed 31 Jan 2022.

[41] Genetic testing reference material coordination program (GeT-RM). https://www.coriell.org/1/NIGMS/Additional-Resources/Multiply-Confirmed-Mutations-GeT-RM. Accessed 31 Jan 2022.

[42] GRCh38 Reference Genome. ftp://ftp.1000genomes.ebi.ac.uk/vol1/ftp/technical/reference/GRCh38_reference_genome/. Accessed 31 Jan 2022.

[43] Institutional Review Board (IRB) of the All of Us Research Program. (2020). https://allofus.nih.gov/about/who-we-are/institutional-review-board-irb-of-all-of-us-research-program. Accessed 31 Jan 2022.

