# Supplementary material for: Whole-genome sequencing as an investigational device for return of hereditary disease risk and pharmacogenomic results as part of the All of Us Research Program
Source: Genome Med. 2022 Mar 28;14:34. doi: 10.1186/s13073-022-01031-z (PMC8962531; doi:10.1186/s13073-022-01031-z)
Supplement: Supplementary file 1 — Additional file 1. Supplementary Tables S1-S25 and Supplementary Figures S1-S8. [file 13073_2022_1031_MOESM1_ESM.docx]

Table S1. Reportable HDR and PGx genes

| **Gene** | **Drug (brand name) / Disease (MIM number)** | **Report type** |
| --- | --- | --- |
| *TPMT* | azathioprine (Imuran®)  mercaptopurine (Purinethol®)  thioguanine | PGx |
| *NUDT15* | azathioprine (Imuran®)  mercaptopurine (Purinethol®)  thioguanine | PGx |
| *DPYD* | capecitabine (Xeloda®)  fluorouracil (Adrucil®) | PGx |
| *UGT1A1* | atazanavir (Reyataz®)  belinostat (Beleodaq®)  Irinotecan (Camptosar®) | PGx |
| *SLCO1B1* | simvastatin (Zocor®) | PGx |
| *CYP2C19* | amitriptyline (Elaviil®)  citalopram (Celexa®)  clobazam (Onfi®)  clomipramine (Anafranil®)  clopidogrel (Plavix®)  doxepin (Sinequan®)  escitalopram (Lexapro®)  imipramine (Tofranil®)  setraline (Zoloft®)  trimipramine (Surmontil®)  voriconazole (Vfend®)  flibanserin (Addyi®)  pantoprazole (Protonix®)  brivaracetam (Briviact®) | PGx |
| *G6PD* | dabrafenib (Tafinlar®)  dapsone  hydroxychloroquine (Plaquenil®)  Local anesthetic containing drugs (e.g. articaine, chloroprocaine, lidocaine, mepivacaine, ropivacaine, tetracaine)  mafenide (Sulfamylon®)  methylene blue  nalidixic acid (NegGram®)  nitrofurantoin (Macrobid®, Macrodantin®, Furadentin®)  pegloticase (Krystexxa®)  phenazopyridine  primaquine  probenecid (Col-Benemid®)  rasburicase (Elitek®)  sodium nitrite  sulfacetamide  sulfamethoxazole/trimethoprim (Bactrim®, Septra®)  sulfanilamide  sulfasalazine (Azulfidine®)  tafenoquine (Krintafel®) | PGx |
| *APC* | Adenomatous polyposis coli (MIM 175100) | HDR |
| *MYH11* | Aortic aneurysm, familial thoracic 4 (MIM 132900) | HDR |
| *ACTA2* | Aortic aneurysm, familial thoracic 6 (MIM 611788) | HDR |
| *TMEM43* | Arrhythmogenic right ventricular cardiomyopathy, type 5 (MIM 604400) | HDR |
| *DSP* | Arrhythmogenic right ventricular cardiomyopathy, type 8 (MIM 607450) | HDR |
| *PKP2* | Arrhythmogenic right ventricular cardiomyopathy, type 9 (MIM 609040) | HDR |
| *DSG2* | Arrhythmogenic right ventricular cardiomyopathy, type 10 (MIM 610193) | HDR |
| *DSC2* | Arrhythmogenic right ventricular cardiomyopathy, type 11 (MIM 610476) | HDR |
| *BRCA1* | Breast-ovarian cancer, familial 1 (MIM 604370) | HDR |
| *BRCA2* | Breast-ovarian cancer, familial 2 (MIM 612555) | HDR |
| *SCN5A* | Brugada syndrome 1 (MIM 601144) | HDR |
| *RYR2* | Catecholaminergic polymorphic ventricular tachycardia (MIM 604772) | HDR |
| *LMNA* | Dilated cardiomyopathy 1A (MIM 115200) | HDR |
| *MYBPC3* | Dilated cardiomyopathy 1A (MIM 115200) | HDR |
| *COL3A1* | Ehlers-Danlos syndrome, type 4 (MIM 130050) | HDR |
| *GLA* | Fabry's disease (MIM 301500) | HDR |
| *APOB* | Familial hypercholesterolemia (MIM 143890) | HDR |
| *LDLR* | Familial hypercholesterolemia (MIM 143890) | HDR |
| *MYH7* | Familial hypertrophic cardiomyopathy 1 (MIM 192600) | HDR |
| *TPM1* | Familial hypertrophic cardiomyopathy 3 (MIM 115196) | HDR |
| *MYBPC3* | Familial hypertrophic cardiomyopathy 4 (MIM 115197) | HDR |
| *PRKAG2* | Familial hypertrophic cardiomyopathy 6 (MIM 600858) | HDR |
| *TNNI3* | Familial hypertrophic cardiomyopathy 7 (MIM 613690) | HDR |
| *MYL3* | Familial hypertrophic cardiomyopathy 8 (MIM 608751) | HDR |
| *MYL2* | Familial hypertrophic cardiomyopathy 10 (MIM 608758) | HDR |
| *ACTC1* | Familial hypertrophic cardiomyopathy 11 (MIM 612098) | HDR |
| *RET* | Familial medullary thyroid carcinoma (MIM 155240) | HDR |
| *PCSK9* | Hypercholesterolemia, autosomal dominant, 3 (MIM 603776) | HDR |
| *BMPR1A* | Juvenile polyposis syndrome, (MIM 174900) | HDR |
| *SMAD4* | Juvenile polyposis syndrome, (MIM 174900) | HDR |
| *TNNT2* | Left ventricular noncompaction 6 (MIM 601494) | HDR |
| *TP53* | Li-Fraumeni syndrome 1 (MIM 151623) | HDR |
| *TGFBR1* | Loeys-Dietz syndrome type 1A (MIM 609192) | HDR |
| *TGFBR2* | Loeys-Dietz syndrome type 1B (MIM 610168) | HDR |
| *TGFBR1* | Loeys-Dietz syndrome type 2A (MIM 608967) | HDR |
| *TGFBR2* | Loeys-Dietz syndrome type 2B (MIM 610380) | HDR |
| *SMAD3* | Loeys-Dietz syndrome type 3 (MIM 613795) | HDR |
| *KCNQ1* | Long QT syndrome 1 (MIM 192500) | HDR |
| *KCNH2* | Long QT syndrome 2 (MIM 613688) | HDR |
| *SCN5A* | Long QT syndrome 3 (MIM 603830) | HDR |
| *MLH1* | Lynch syndrome (MIM 120435) | HDR |
| *MSH2* | Lynch syndrome (MIM 120435) | HDR |
| *MSH6* | Lynch syndrome (MIM 120435) | HDR |
| *PMS2* | Lynch syndrome (MIM 120435) | HDR |
| *RYR1* | Malignant hyperthermia (MIM 145600) | HDR |
| *CACNA1S* | Malignant hyperthermia (MIM 145600) | HDR |
| *FBN1* | Marfan's syndrome (MIM 154700) | HDR |
| *TGFBR1* | Marfan's syndrome (MIM 154700) | HDR |
| *MEN1* | Multiple endocrine neoplasia, type 1 (MIM 131100) | HDR |
| *RET* | Multiple endocrine neoplasia, type 2a (MIM 171400) | HDR |
|  | Multiple endocrine neoplasia, type 2b (MIM 162300) | HDR |
| *MUTYH* | MYH-associated polyposis (MIM 608456) | HDR |
| *NF2* | Neurofibromatosis, type 2 (MIM 101000) | HDR |
| *OTC* | Ornithine carbamoyltransferase deficiency (MIM 311250) | HDR |
| *SDHD* | Paragangliomas 1 (MIM 168000) | HDR |
| *SDHAF2* | Paragangliomas 2 (MIM 601650) | HDR |
| *SDHC* | Paragangliomas 3 (MIM 605373) | HDR |
| *SDHB* | Paragangliomas 4 (MIM 115310) | HDR |
| *STK11* | Peutz-Jeghers syndrome (MIM 175200) | HDR |
| *MUTYH* | Pilomatrixoma (MIM 132600) | HDR |
| *PTEN* | PTEN hamartoma tumor syndrome (MIM 153480) | HDR |
| *RB1* | Retinoblastoma (MIM 180200) | HDR |
| *TSC1* | Tuberous sclerosis 1 (MIM 191100) | HDR |
| *TSC2* | Tuberous sclerosis 2 (MIM 613254) | HDR |
| *VHL* | Von Hippel-Lindau syndrome (MIM 193300) | HDR |
| *WT1* | Wilms' tumor (MIM 194070) | HDR |
| *ATP7B* | Wilson disease (MIM 277900) | HDR |

Table S2. Founder mutations in *BRCA1* and *BRCA2* called with 100% accuracy in independent samples

| **Sample ID** | **Gene** | **DNA change** | **Amino acid change** | **GRCh37 coordinates** | **Variant type** | **Zygosity** | **Site** |
| --- | --- | --- | --- | --- | --- | --- | --- |
| 1000070014 | *BRCA1* | c.68_69delAG | p.Glu23Valfs*17 | 17:41276045_41276046del | Deletion | Heterozygous | BCM*^a^* |
| 1000073979 | *BRCA1* | c.5266dupC | p.Gln1756Profs*74 | 17:41209080dupC | Insertion | Heterozygous | BCM |
| SM-IN9H2 | *BRCA1* | c.68_69delAG | p.Glu23Valfs*17 | 17:41276045_41276046delCT | Deletion | Heterozygous | BI*^b^* |
| SM-IN8QQ | *BRCA1* | c.5266dupC | p.Gln1756Profs*74 | 17:41209079_41209080insG | Insertion | Heterozygous | BI |
| SM-JPV8J | *BRCA1* | c.5266dupC | p.Gln1756Profs*74 | 17:41209079T>TG | Insertion | Heterozygous | BI |
| SM-JPV8Y | *BRCA1* | c.5266dupC | p.Gln1756Profs*74 | 17:41209079T>TG | Deletion | Heterozygous | BI |
| SM-JPVA4 | *BRCA1* | c.5266dupC | p.Gln1756Profs*74 | 17:41209079T>TG | Insertion | Heterozygous | BI |
| SM-JPVAE | *BRCA1* | c.5266dupC | p.Gln1756Profs*74 | 17:41209079T>TG | Insertion | Heterozygous | BI |
| 1000074456 | *BRCA2* | c.5946delT | p.Ser1982Argfs*22 | 13:32914438del | Deletion | Heterozygous | BCM |
| SM-GZQK6 | *BRCA2* | c.5946delT | p.Ser1982Argfs*22 | 13:32914438delT | Deletion | Heterozygous | BI |
| SM-JPTV9 | *BRCA2* | c.5946delT | p.Ser1982Argfs*22 | 13:32914437GT>G | Deletion | Heterozygous | BI |
| SM-JPV89 | *BRCA2* | c.5946delT | p.Ser1982Argfs*22 | 13:32914437GT>G | Deletion | Heterozygous | BI |
| SM-JPVA9 | *BRCA2* | c.5946delT | p.Ser1982Argfs*22 | 13:32914437GT>G | Deletion | Heterozygous | BI |
| 323249 | *BRCA2* | c.5946delT | p.Ser1982Argfs*22 | 13:32914438delT | Deletion | Heterozygous | UW*^c^* |

*^a^*BCM, Baylor College of Medicine. *^b^*BI, Broad Institute. *^c^*UW, University of Washington.

Table S3. Call concordance of select pathogenic variants in human cell lines


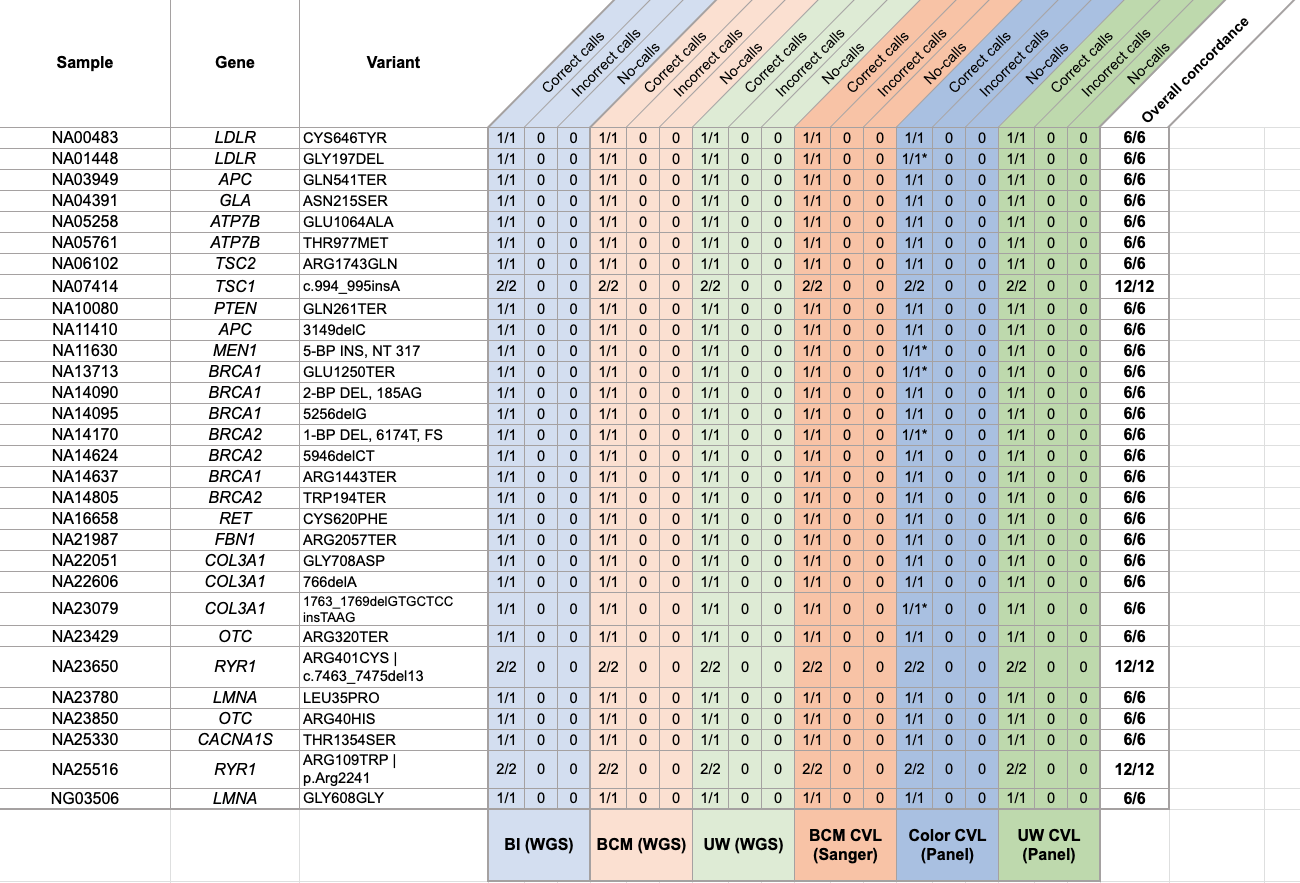


Samples marked with asterisks were run in triplicate at one site. All calls between replicates were identical. BI, Broad Institute. WGS, whole genome sequencing. BCM, Baylor College of Medicine. UW, University of Washington. CVL, Clinical Validation Laboratory. Color, Color Health.

Table S4. Call concordance of PGx alleles using WGS and orthogonal methods in GeT-RM cell lines


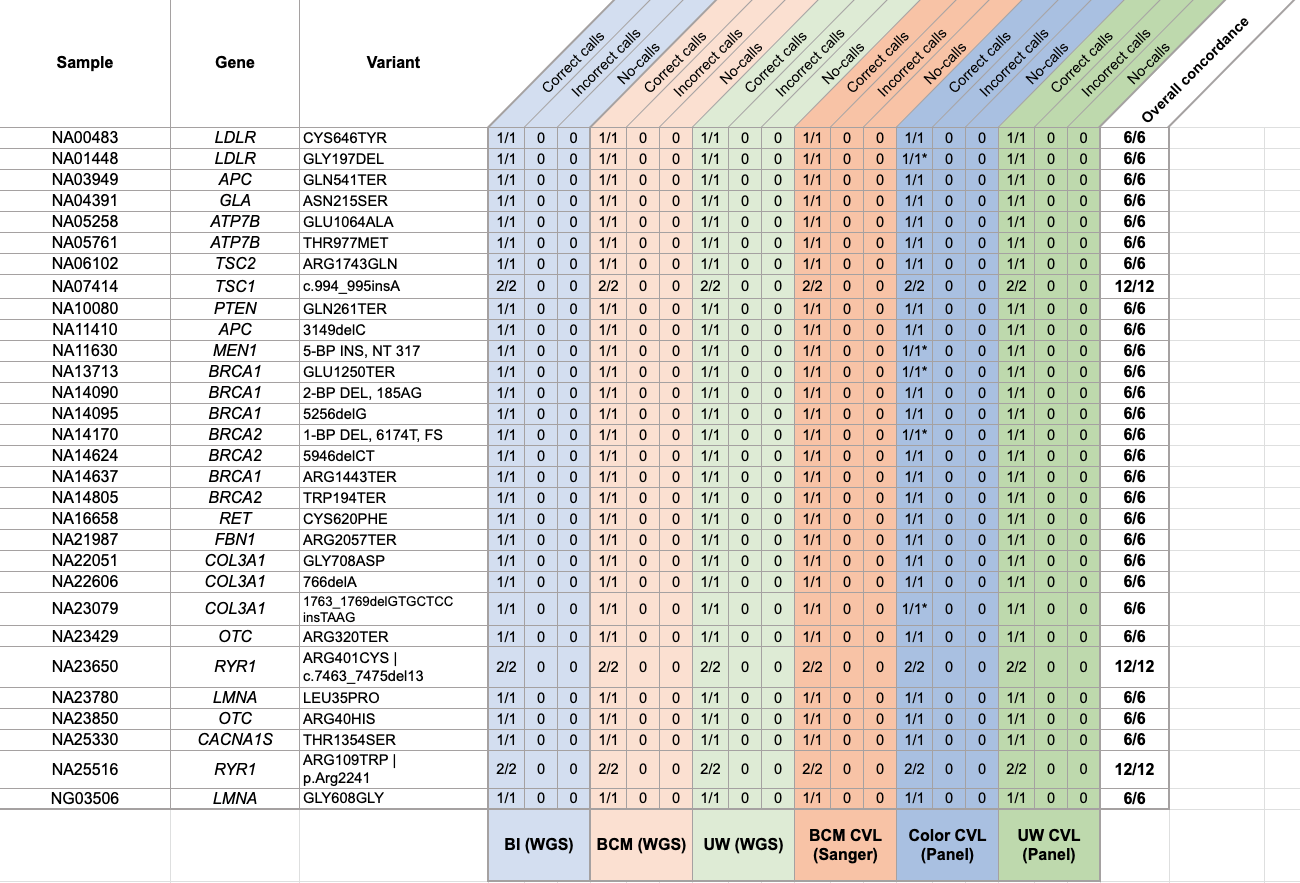


PGx, pharmacogenomics. BCM, Baylor College of Medicine. UW, University of Washington. Color, Color Health.

Table S5. PGx calling across additional cell line samples from the 1000 Genomes Project

| **Gene and star allele** | **PGx***^a^* **sample** | **Number of samples** | **Correct calls** | **Incorrect calls** | **Overall concordance** |
| --- | --- | --- | --- | --- | --- |
| *CYP2C19*16* | NA19452 | 1 | 1/1 | 0 | 1/1 |
| *CYP2C19*2* | HG02087 HG02511 NA18599 NA18617 NA18964 NA18978 NA18998 NA19000 NA19020 NA21110 | 10 | 10/10 | 0 | 10/10 |
| *CYP2C19*2/*2* | HG02186 HG02188 | 2 | 2/2 | 0 | 2/2 |
| *CYP2C19*22* | HG02318 | 1 | 1/1 | 0 | 1/1 |
| *CYP2C19*24* | NA20356 | 1 | 1/1 | 0 | 1/1 |
| *CYP2C19*3* | HG02141 HG02318 | 2 | 2/2 | 0 | 2/2 |
| *G6PD* Asahi | HG02511 | 1 | 1/1 | 0 | 1/1 |
| *G6PD* Aures | HG02165 | 1 | 1/1 | 0 | 1/1 |
| *G6PD* Canton, Taiwan-Hakka, Gifu-like, Agrigento-like | HG02087 NA18617 | 2 | 2/2 | 0 | 2/2 |
| *G6PD* Chinese-5 | HG02186 | 1 | 1/1 | 0 | 1/1 |
| *G6PD* Ilesha | NA19020 | 1 | 1/1 | 0 | 1/1 |
| *G6PD* Kalyan-Kerala, Jamnaga, Rohini | NA21110 | 1 | 1/1 | 0 | 1/1 |
| *G6PD* Seattle, Lodi, Modena, Ferrara-II, Athens-like | HG01620 | 1 | 1/1 | 0 | 1/1 |
| *G6PD* Sibari | NA19323 | 1 | 1/1 | 0 | 1/1 |
| *G6PD* Ube Konan | NA18998 | 1 | 1/1 | 0 | 1/1 |
| *G6PD* Viangchan, Jammu | HG02141 HG02188 | 2 | 2/2 | 0 | 2/2 |
| *NUDT15*2* | NA18599 NA18621 NA18622 NA18626 NA18633 NA18740 NA18964 NA18978 NA19000 NA19077 | 10 | 10/10 | 0 | 10/10 |
| *NUDT15*3* | NA18599 NA18998 | 2 | 2/2 | 0 | 2/2 |
| *SLCO1B1*15* | NA19000 | 1 | 1/1 | 0 | 1/1 |
| *SLCO1B1*17* | HG02186 HG02188 NA18599 NA19000 NA19077 | 5 | 5/5 | 0 | 5/5 |
| *TPMT*2* | HG01605 | 1 | 1/1 | 0 | 1/1 |
| *TPMT*3C* | NA18908 NA19114 NA19323 NA19452 | 4 | 4/4 | 0 | 4/4 |
| *UGT1A1*28* | NA18510 NA18908 NA19077 NA19323 | 4 | 4/4 | 0 | 4/4 |
| *UGT1A1*6* | HG02141 HG02188 NA18617 NA18621 NA18626 NA18633 NA18740 | 7 | 7/7 | 0 | 7/7 |

*^a^*PGx, pharmacogenomics.

Table S6. Overall equivalence of called variants in donor blood samples across the Genome Centers

|  |  | **SNV***^d^* **concordant** | **SNV discordant** | **InDel***^e^* **concordant** | **InDel discordant** | **Overall equivalence [95%CI***^f^***]** |
| --- | --- | --- | --- | --- | --- | --- |
| **Interlab** | **BCM***^a^***-UW***^b^* | 3830/3833 | 3/3833 | 189/195 | 6/195 | 99.77%  [99.55%-99.99%] |
|  | **BI***^c^***-BCM** | 3830/3834 | 4/3834 | 191/193 | 2/193 | 99.86%  [99.71%-100%] |
|  | **UW-BI** | 3831/3834 | 3/3834 | 188/196 | 8/196 | 99.71%  [99.46%-99.96%] |
| **Intralab** | **BI-BI** | 1708/1709 | 1/1709 | 87/89 | 2/89 | 99.82%  [99.57%-100%] |
|  | **BCM-BCM** | 1246/1248 | 2/1248 | 62/62 | 0/62 | 99.76%  [99.32%-100%] |
|  | **UW-UW** | 1577/1578 | 1/1578 | 76/83 | 7/83 | 99.49%  [99.02%-99.46%] |

*^a^*BCM, Baylor College of Medicine. *^b^*UW, University of Washington. *^c^*BI, Broad Institute. *^d^*SNV, single nucleotide variant. *^e^*InDel, insertion or deletion. *^f^*CI, Confidence interval.

Table S7. Overall equivalence of called variants in cell lines across the Genome Centers

|  |  | **SNV***^d^* **Concordant** | **SNV discordant** | **InDel***^e^* **concordant** | **InDel discordant** | **Overall equivalence [95%CI***^f^***]** |
| --- | --- | --- | --- | --- | --- | --- |
| **Interlab** | **BCM***^a^***-UW***^b^* | 24289/24304 | 15/24304 | 1142/1178 | 36/1178 | 99.80%  [99.71%-99.89%] |
|  | **BI***^c^***-BCM** | 24288/24304 | 16/24304 | 1151/1171 | 20/1171 | 99.86%  [99.79%-99.93%] |
|  | **UW-BI** | 24287/24302 | 15/24302 | 1145/1177 | 32/1177 | 99.81%  [99.73%-99.90%] |
| **Intralab** | **BI-BI** | 1175/1175 | 0/1175 | 56/65 | 9/65 | 99.27%  [99.68%-99.86%] |
|  | **BCM-BCM** | 806/806 | 0/806 | 38/40 | 2/40 | 99.76%  [99.32%-100%] |
|  | **UW-UW** | 918/918 | 0/918 | 50/50 | 0/50 | 100%  [100%-100%] |

*^a^*BCM, Baylor College of Medicine. *^b^*UW, University of Washington. *^c^*BI, Broad Institute. *^d^*SNV, single nucleotide variant. *^e^*InDel, insertion or deletion. *^f^*CI, confidence interval.

Table S8. Concordance of variant calling by genomic context in donor blood samples

| **Category** | **Genome Center** | **Panel+/WGS–**  **(false neg.)** | **Panel– /WGS+**  **(false pos.)** | **Panel+ /WGS+**  **(true pos.)** | **Panel– /WGS–**  **(true neg.)** | **PPA***^c^* **[95% CI***^d^***]** | **NPA***^e^* **[95% CI]** |
| --- | --- | --- | --- | --- | --- | --- | --- |
| **SNVs***^f^* | BCM*^g^* | 7 | 13 | 2609 | 4187271 | 99.74% [99.6%-99.9%] | 100% [100%-100%] |
|  | BI*^h^* | 8 | 14 | 2610 | 4187268 | 99.71% [99.5%-99.9%] | 100% [100%-100%] |
|  | UW*^i^* | 8 | 13 | 2610 | 4187269 | 99.71% [99.5%-99.9%] | 100% [100%-100%] |
| **Insertions** | BCM | 1 | 20 | 20 | 4189859 | 97.22% [91.8%-100%] | 100% [100%-100%] |
|  | BI | 0 | 20 | 20 | 4189860 | 100% [100%-100%] | 100% [100%-100%] |
|  | UW | 2 | 20 | 20 | 4189858 | 94.44% [87.1%-100%] | 100% [100%-100%] |
| **Deletions** | BCM | 1 | 0 | 42 | 4189857 | 98.33% [95.1%-100%] | 100% [100%-100%] |
|  | BI | 1 | 0 | 42 | 4189857 | 98.33% [95.1%-100%] | 100% [100%-100%] |
|  | UW | 1 | 0 | 42 | 4189857 | 98.33% [95.1%-100%] | 100% [100%-100%] |
| **Segmental duplications** | BCM | 1 | 0 | 240 | 4189659 | 99.29% [97.9%-100%] | 100% [100%-100%] |
|  | BI | 1 | 0 | 240 | 4189659 | 99.29% [97.9%-100%] | 100% [100%-100%] |
|  | UW | 1 | 0 | 240 | 4189659 | 99.29% [97.9%-100%] | 100% [100%-100%] |
| **Low mappability regions** | BCM | 1 | 0 | 24 | 3770885 | 97.22% [91.8%-100%] | 100% [100%-100%] |
|  | BI | 1 | 0 | 24 | 3770885 | 97.22% [91.8%-100%] | 100% [100%-100%] |
|  | UW | 1 | 0 | 24 | 3770885 | 97.22% [91.8%-100%] | 100% [100%-100%] |
| **Low complexity**  **regions** | BCM | 0 | 0 | 36 | 4189864 | 100% [100%-100%] | 100% [100%-100%] |
|  | BI | 0 | 0 | 36 | 4189864 | 100% [100%-100%] | 100% [100%-100%] |
|  | UW | 0 | 0 | 36 | 4189864 | 100% [100%-100%] | 100% [100%-100%] |
| **Low GC regions** | BCM | 0 | 0 | 20 | 1675940 | 100% [100%-100%] | 100% [100%-100%] |
|  | BI | 0 | 0 | 20 | 1675940 | 100% [100%-100%] | 100% [100%-100%] |
|  | UW | 0 | 0 | 20 | 1675940 | 100% [100%-100%] | 100% [100%-100%] |
| **Heterozygous variants** | BCM | 11 | 15 | 1648 | 4188226 | 99.37% [99.0%-99.7%] | 100% [100%-100%] |
|  | BI | 13 | 16 | 1648 | 4188223 | 99.27% [98.9%-99.7%] | 100% [100%-100%] |
|  | UW | 13 | 15 | 1648 | 4188224 | 99.27% [98.9%-99.7%] | 100% [100%-100%] |
| **Homozygous variants** | BCM | 1 | 18 | 1025 | 4188856 | 99.92% [99.8%-100%] | 100% [100%-100%] |
|  | BI | 0 | 18 | 1026 | 4188856 | 100% [100%-100%] | 100% [100%-100%] |
|  | UW | 0 | 18 | 1026 | 4188856 | 100% [100%-100%] | 100% [100%-100%] |

The data presented here come from a set of 20 donor blood samples from five individuals. Each sample was sequenced using both an existing, validated clinical sequencing gene panel test and the WGS-based test that will be used for the AoURP. Using vcfeval, we then compared the WGS data to the clinical gene panel data and assessed performance on a variety of genomic contexts. Panel+ indicates that a variant genotype was present on the clinical gene panel, while Panel- means the variant genotype was not present on the Panel. Similarly, WGS+ means the variant genotype was present in the WGS data, while WGS- means it was absent. *^c^*PPA, positive percent agreement. *^d^*CI, confidence interval. *^e^*NPA, negative percent agreement. *^f^*SNVs, single nucleotide variants. *^g^*BCM, Baylor College of Medicine. *^h^*BI, Broad Institute. *^i^*UW, University of Washington.

Table S9. Concordance of variant calling by genomic context in cell lines

| **Category** | **Genome Center** | **Panel+***^a^***/WGS–***^b^* | **Panel– /WGS+** | **Panel+ /WGS+** | **Panel– /WGS–** | **PPA***^c^* **[95% CI***^d^***]** | **NPA***^e^* **[95% CI]** |
| --- | --- | --- | --- | --- | --- | --- | --- |
| **SNVs***^f^* | BCM*^g^* | 24 | 1 | 3558 | 6071772 | 99.34% [99.0%-99.7%] | 100% [100%-100%] |
|  | BI*^h^* | 24 | 1 | 3694 | 6281131 | 99.36% [99.1%-99.7%] | 100% [100%-100%] |
|  | UW*^i^* | 24 | 0 | 3694 | 6281132 | 99.36% [99.1%-99.7%] | 100% [100%-100%] |
| **Insertions** | BCM | 1 | 23 | 21 | 5237330 | 96.70% [90.1%-100%] | 100% [100%-100%] |
|  | BI | 1 | 22 | 22 | 5446825 | 96.88% [90.8%-100%] | 100% [100%-100%] |
|  | UW | 1 | 23 | 22 | 5446824 | 96.88% [90.8%-100%] | 100% [100%-100%] |
| **Deletions** | BCM | 1 | 9 | 63 | 5865787 | 99.11% [97.4%-100%] | 100% [100%-100%] |
|  | BI | 1 | 9 | 65 | 6075280 | 99.14% [97.4%-100%] | 100% [100%-100%] |
|  | UW | 1 | 10 | 65 | 6075279 | 99.14% [97.4%-100%] | 100% [100%-100%] |
| **Segmental duplications** | BCM | 0 | 0 | 309 | 6075046 | 100% [100%-100%] | 100% [100%-100%] |
|  | BI | 0 | 1 | 322 | 6284527 | 100% [100%-100%] | 100% [100%-100%] |
|  | UW | 0 | 0 | 322 | 6284528 | 100% [100%-100%] | 100% [100%-100%] |
| **Low mappability regions** | BCM | 0 | 0 | 16 | 2932915 | 100% [100%-100%] | 100% [100%-100%] |
|  | BI | 0 | 0 | 18 | 3142407 | 100% [100%-100%] | 100% [100%-100%] |
|  | UW | 0 | 0 | 18 | 3142407 | 100% [100%-100%] | 100% [100%-100%] |
| **Low complexity regions** | BCM | 0 | 11 | 54 | 6075290 | 100% [100%-100%] | 100% [100%-100%] |
|  | BI | 0 | 11 | 54 | 6075290 | 100% [100%-100%] | 100% [100%-100%] |
|  | UW | 0 | 10 | 54 | 6075291 | 100% [100%-100%] | 100% [100%-100%] |
| **Low GC regions** | BCM | 1 | 0 | 34 | 4399360 | 99.21% [97.7%-100%] | 100% [100%-100%] |
|  | BI | 1 | 0 | 36 | 4608853 | 99.24% [97.8%-100%] | 100% [100%-100%] |
|  | UW | 1 | 0 | 36 | 4608853 | 99.24% [97.8%-100%] | 100% [100%-100%] |
| **Heterozygous variants** | BCM | 18 | 20 | 2225 | 6073092 | 99.22% [98.7%-99.7%] | 100% [100%-100%] |
|  | BI | 18 | 20 | 2323 | 6282489 | 99.24% [98.8%-99.7%] | 100% [100%-100%] |
|  | UW | 18 | 20 | 2323 | 6282489 | 99.24% [98.8%-99.7%] | 100% [100%-100%] |
| **Homozygous variants** | BCM | 8 | 13 | 1417 | 6073917 | 99.52% [99.1%-100%] | 100% [100%-100%] |
|  | BI | 8 | 12 | 1458 | 6283372 | 99.54% [99.1%-100%] | 100% [100%-100%] |
|  | UW | 8 | 13 | 1458 | 6283371 | 99.54% [99.1%-100%] | 100% [100%-100%] |

*^a^+* indicates that a variant was present on the panel or the corresponding whole genome sequencing (WGS) sample. *^b^*– indicates that a variant was not present on the panel or the corresponding WGS sample. *^c^*PPA, positive percent agreement. *^d^*CI, confidence interval. *^e^*NPA, negative percent agreement. *^f^*SNVs, single nucleotide variants. *^g^*BCM, Baylor College of Medicine. *^h^*BI, Broad Institute. *^i^*UW, University of Washington.

Table S10. Equivalence of performance measures between cell line-derived DNA and blood-derived DNA

| **Performance measures** | | | | | | | | | | |
| --- | --- | --- | --- | --- | --- | --- | --- | --- | --- | --- |
|  | **Accuracy (n)** | **P/LP***^a^* **variant accuracy** | **PGx***^b^* **allele accuracy** | **Precision (n)** | **Interlab concordance (BCM***^c^***-UW***^d^***) [95% CI***^e^***]** | | **Interlab concordance (BI***^f^***-BCM) [95% CI]** | | **Interlab concordance (UW-BI) [95% CI]** | |
| **Clinical samples** | 271 | 100% | 100% | 28 | 99.77%  [99.55%-99.99%] | | 99.86%  [99.71%-100%] | | 99.71%  [99.46%-99.96%] | |
| **Cell line samples** | 30 | 100% | 100% | 175 | 99.8%  [99.71%-99.89%] | | 99.86%  [99.79%-99.93%] | | 99.81%  [99.73%-99.90%] | |

*^a^*Pathogenic/likely pathogenic. *^b^*PGx, pharmacogenomics. *^c^*BCM, Baylor College of Medicine. *^d^*UW, University of Washington. *^f^*CI, confidence interval. *^f^*BI, Broad Institute.

Table S11. Equivalence technical metrics between cell line-derived DNA and blood-derived DNA

| **Technical metrics** | | | | | | | | | | |
| --- | --- | --- | --- | --- | --- | --- | --- | --- | --- | --- |
|  | **Number of samples** | **% aligned bases**  **[std dev***^a^***]** | **% duplicate reads**  **[std dev]** | **Insert size (bp)**  **[std dev]** | **% Q30 bases**  **[std dev]** | **% chimeric reads**  **[std dev]** | **Genome coverage [std dev]** | **% covered ≥20X**  **[std dev]** | **% contam- ination**  **[std dev]** | **Ti/Tv ratio**  **[std dev]** |
|  |  |  |  |  |  |  |  |  |  |  |
| **Clinical samples** | 253 | 92%  [±0.94%] | 11.15%  [±1.78%] | 421 bp  [±16 bp] | 92.2%  [±0.77%] | 1.81%  [±0.24%] | 39.99X  [±7.3X] | 96.55%  [±0.49%] | 0.03%  [±0.33%] | 1.94  [±0.01] |
| **Cell line samples** | 223 | 91.25%  [±0.93%] | 11.14%  [±1.4%] | 419 bp  [±11 bp] | 91.43%  [±1.03%] | 1.69%  [±0.16%] | 39.53X  [±3.13X] | 96.07%  [±0.24%] | 0.02%  [±0.07%] | 1.93  [± 0.01] |

*^a^*Std dev, standard deviation.

Table S12. Concordance of PGx calling across the Genome Centers


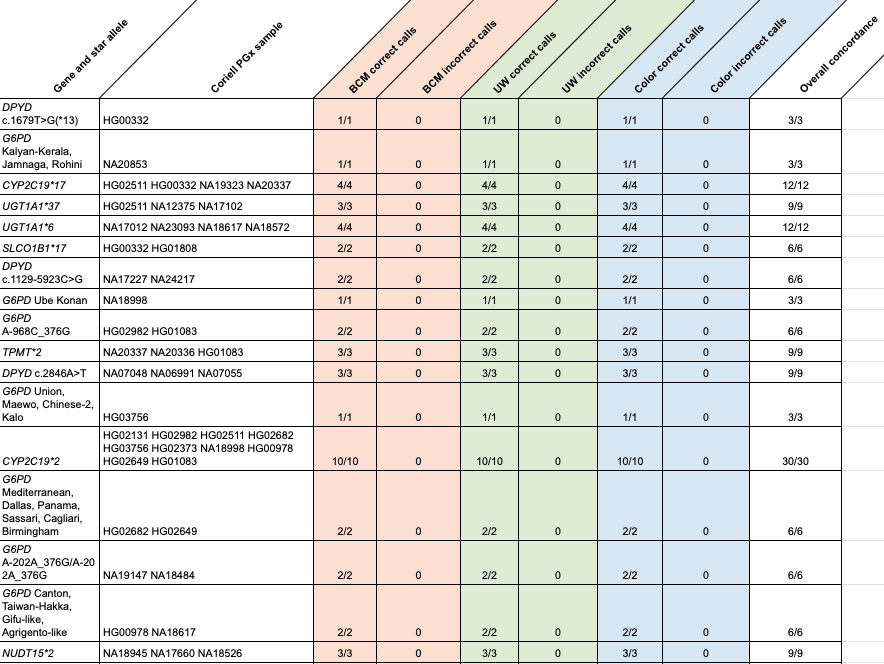


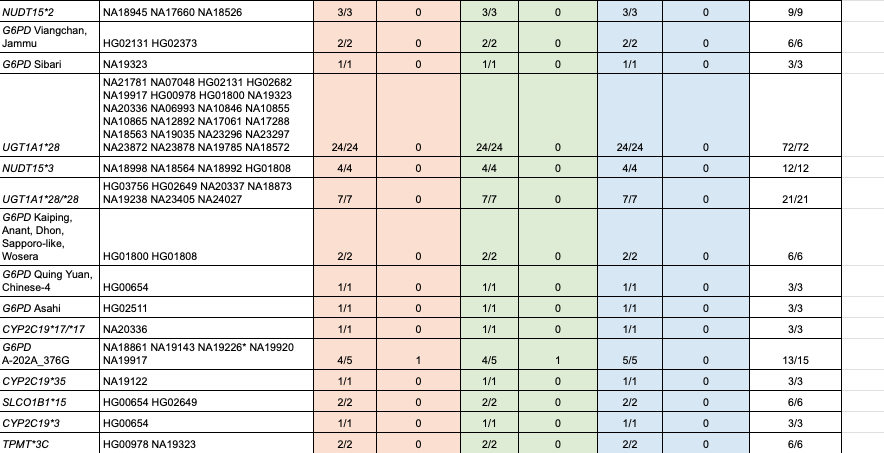


PGx, pharmacogenomics. BCM, Baylor College of Medicine. UW, University of Washington.

Table S13. Input titration results for four blood donor samples

| **Input (ng)** | **Library construction success rate** | **Mean sensitivity** | **Std dev***^a^* **sensitivity** | **Mean precision** | **Std dev precision** |
| --- | --- | --- | --- | --- | --- |
| 25 | 0% | n/a^b^ | n/a | n/a | n/a |
| 100 | 17% | 99% | n/a | 100% | n/a |
| 250 | 100% | 100% | 0% | 100% | 0% |
| 375 | 100% | 98% | 1% | 99% | 0% |
| 500 | 100% | 99% | 1% | 99% | 1% |
| 750 | 100% | 98% | 1% | 99% | 0% |
| 1500 | 100% | 98% | 1% | 98% | 0% |

*^a^*Std dev, standard deviation.^b^n/a = not applicable due to no data or not enough data.

Table S14. Details of variants excluded due to repeats and homopolymers

| **chr** | **start** | **end** | **gene** | **bases excluded** | **genomic context (intersection w/ GA4GH bed)** |
| --- | --- | --- | --- | --- | --- |
| 2 | 47641528 | 47641541 | MSH2 | 13 | GC<25% (2:47641528-47641541) |
| 7 | 150647249 | 150647262 | KCNH2 | 13 | segmental duplication (7:150647253-150647262) |
| 7 | 150674919 | 150674928 | KCNH2 | 9 | NA |
| 7 | 150674952 | 150674962 | KCNH2 | 10 | NA |
| 9 | 135786961 | 135786970 | TSC1 | 9 | NA |
| 16 | 15857646 | 15857651 | MYH11 | 5 | segmental duplication (16:15857649-15857651) |

Table S15. Frequently underperforming bases within the Hereditary Disease Risk Report

| **Gene** | **Total sites** | **GRCh37**  **low-coverage sites** | **GRCh38**  **low-coverage sites** |
| --- | --- | --- | --- |
| *MYH11* | 6699 | 8 | 8 |
| *MSH2* | 3148 | 14 | 14 |
| *KCNH2* | 3131 | 24 | 23 |
| *TSC1* | 3936 | 10 | 10 |

Table S16. Fail rates for samples at discrete parts of the process across the Genome Centers

| **Genome Centers (WGS***^a^***)** | | | | | | | | | | | | |
| --- | --- | --- | --- | --- | --- | --- | --- | --- | --- | --- | --- | --- |
| **Lab** | **Cohort** | **Instrument** | **Sample intake** | | **Library** | | **Sequencing** | | **Data quality** | | **Total samples failed** | **Aggregate sample fail rate** |
|  |  |  | **Input #** | **# failed (% of total fails)** | **Input #** | **# failed (% of total fails)** | **Input #** | **# failed (% of total fails)** | **Input #** | **# failed (% of total fails)** |  |  |
| **BCM***^b^* | TOPMed*^f^* | NovaSeq | 1986 | 103 (90.4%) | 1883 | 0 (0%) | 1883 | 0 (0%) | 1883 | 11 (9.6%) | 114 | 5.74% |
|  | AoURP*^g^* | NovaSeq | 584 | 0 (0%) | 584 | 0 (0%) | 584 | 0 (0%) | 584 | 1 (100%) | 1 | 0.17% |
| **Broad***^c^* | TOPMed | NovaSeq | 6391 | 547 (99.5%) | 5844 | 3 (0.5%) | 5841 | 0 (0%) | 5841 | 0 (0%) | 550 | 8.61% |
|  | AoURP | NovaSeq | 424 | 3 (33.3%) | 421 | 0 (0%) | 421 | 0 (0%) | 421 | 6 (66.7%) | 9 | 2.12% |
| **UW***^d^* | TOPMed | HiSeqX | 14587 | 1346 (88.3%) | 13241 | 45 (3%) | 13196 | 102 (6.7%) | 13094 | 32 (2%) | 1,525 | 10.45% |
|  | AoURP | NovaSeq | 291 | 0 (0%) | 291 | 0 (0%) | 291 | 2 (33.3%) | 289 | 4 (66.7%) | 6 | 2.06% |

*^a^*WGS, whole genome sequencing. *^b^*Baylor College of Medicine. *^c^*Broad Institute. *^d^*University of Washington. *^e^*BCM sample fails are in amplicon generation. *^f^*National Heart, Lung, and Blood Institute Trans-Omics for Precision Medicine. *^g^All of Us* Research Program.

Table S17. Fail rates for samples at discrete parts of the process across the Clinical Validation Laboratories

| **CVLs***^a^* | | | | | | | | | | | | |
| --- | --- | --- | --- | --- | --- | --- | --- | --- | --- | --- | --- | --- |
| **Lab** | **Cohort** | **Instrument** | **Sample Intake** | | **Library** | | **Sequencing** | | **Data Quality** | | **Total samples failed** | **Aggregate sample fail rate** |
|  |  |  | **Input #** | **# Failed (% of total fails)** | **Input #** | **# Failed (% of total fails)** | **Input #** | **# failed (% of total fails)** | **Input #** | **# failed (% of total fails)** |  |  |
| **BCM***^b^* | Internal | ABI 3730/3500 | 1635 | 0 (0%) | NA | NA | 1635 | 0 (0%) | 1635 | 0 (0%) | 10*^d^* | 0.61% |
| **Color** | AoURP*^d^* | NovaSeq | 114 | 0 (0%) | 114 | 1 (100%) | 113 | 0 (0%) | 113 | 0 (0%) | 1 | 0.88% |
| **UW***^c^* | AoURP | NovaSeq | 315 | 0 (0%) | 315 | 0 (0%) | 315 | 2 (50%) | 313 | 2 (50%) | 4 | 1.27% |

*^a^*CVLs, Clinical Validation Laboratories.*^b^*BCM, Baylor College of Medicine. *^c^*UW, University of Washington. *^d^All of Us* Research Program.

Table S18. Feedback post IDE submission

| **Feedback area** | **Post-submission United States Food and Drug Administration feedback** |
| --- | --- |
| Program organization | Please provide a description of the enrollment, informed consent, and biospecimen collection procedures. |
|  | Clarify whether all Clinical Validation Laboratories have the same roles. |
|  | Please describe any processes/procedures in place to ensure that bias is not introduced during the resolution of variant harmonization disagreements. |
|  | Please provide the informing loop information that will be provided to subjects for our review. |
|  | Please provide a detailed description of your procedures/processes for updating variant classifications. |
|  | Describe how data will be delivered to participants, including details about the participant portal interface. |
|  | Please provide all educational materials that will be provided/available to subjects for our review. |
|  | Please clarify in which situations the genetic counselling resource will provide support and the level of support that will be provided, particularly in the case of a significant re-classification in either direction. |
| Report design / language | You should include in your reports that there is a possibility that the variant interpretation of the test result provided by your investigational device is incorrect. |
|  | Do not include any language that may imply to a subject that the research result from your investigational device is actionable. |
|  | Provide the report language that will be used to describe a positive findinghg in each reportable gene. |
| Study design | The United States Food and Drug Administration considers the study investigational in nature (i.e., results from your study are not generated from a cleared or approved assay validated for clinical use). Revise your protocol to consistently convey that your study is an investigational device study. |
|  | All risks of the study should be conveyed together in the consent. |
|  | Please clarify the reporting process for subjects who are Hereditary Disease Risk positive for multiple genes. |
|  | Clarify what raw data types the participants may elect to receive. |
|  | For pharmacogenomic gene-drug associations, do not report associations with ‘moderate’ evidence. |
| Technical information | Please provide a description of parameter settings for running the bioinformatics pipeline and ensure that each sequencing center is using the same parameter. These parameters should be locked throughout the investigational device study |
| Validation study requirements | Provide the false negative rate for the device |
|  | Please provide a detailed description of the orthogonal method used to generate ‘ground truth’ in the accuracy study and your justification for why this assay was selected. |
|  | Justify the number of control samples used for *G6PD* Asahi. |
|  | Clarify/explain a specific discordance between the *UGT1A1**28/*28 ground truth and validation data. |
|  | Please clarify if you have evaluated the reproducibility of your investigational device, in terms of reagent lot to lot, instrument to instrument, operator to operator, and day to day reproducibility. |
|  | Please provide protocols or detailed descriptions of the experimental designs and data analysis methods sufficient to understand how the studies were conducted. |
|  | You should include information in your investigational device exemption submission to demonstrate that the alleles you have chosen are adequate to represent the alleles your investigational device is intended to detect. |
|  | If you have a protocol (with acceptance criteria) describing the validation of new genes to be added to your investigational device in the future, please provide it |
|  | If you have any information about substances known to interfere with your device technology and intended sample type, please provide it. |
|  | Please clarify the patient sample types (anticoagulated whole blood, saliva, etc.) used for this study. |
|  | Please provide a breakdown of the user comprehension study results for each survey question by education level (e.g., breakdown of education levels for those who answered incorrectly for each survey question). |
|  | Please describe any discordant results from these precision studies. |

##

Table S19. Excluded recurrent false positive and false negative variants

| **Gene** | **Variant start site** |
| --- | --- |
| *MSH2* | chr2:47641559 |
| *MSH2* | chr2:47641562 |
| *APOB* | chr2:21266774 |
| *PMS2* | chr7:6037057 |
| *TSC1* | chr9:135773000 |
| *PCSK9* | chr1:55505552 |

Table S20. Accuracy of variant calls across the reportable region of NA12878

| **Reportable region** | **TP***^a^* | **TN***^b^* | **FP***^c^* | **FN***^d^* | **PPV***^e^* **[95% CI***^f^***]** | **NPV***^g^* **[95% CI]** |
| --- | --- | --- | --- | --- | --- | --- |
| All variants | 131 | 218551 | 0 | 0 | 100% [97.2% - 100%] | 100% [100% - 100%] |
| SNVs*^h^* only | 129 | 218551 | 0 | 0 | 100% [97.2% - 100%] | 100% [100% - 100%] |
| Insertions and deletions | 2 | 218551 | 0 | 0 | *** | 100% [100% - 100%] |
| Segmental duplications | 12 | 19073 | 0 | 0 | 100% [73.5% - 100%] | 100% [100% - 100%] |
| Low mappability regions | 1 | 3532 | 0 | 0 | *** | 100% [100% - 100%] |
| Low complexity regions | 4 | 2435 | 0 | 0 | *** | 100% [100% - 100%] |
| Low GC regions | 2 | 2722 | 0 | 0 | *** | 100% [100% - 100%] |
| High GC regions | 0 | 7 | 0 | 0 | *** | 100% [100% - 100%] |
| Heterozygous variants only | 82 | 218551 | 0 | 0 | 100% [95.6% - 100%] | 100% [100% - 100%] |
| Homozygous variants only | 49 | 218551 | 0 | 0 | 100% [92.7% -100%] | 100% [100% - 100%] |

*^a^*TP, true positive. *^b^*TN, true negative. *^c^*FP, false positive. *^d^*FN, false negative. *^e^*PPV, positive predictive value. *^f^*CI, confidence interval. *^g^*NPV, negative predictive agreement. *^h^*SNVs, single-nucleotide variants.

Table S21. Accuracy of variant calls across the whole genome of NA12878

| **Whole genome** | **TP***^a^* | **TN***^b^* | **FP***^c^* | **FN***^d^* | **PPV***^e^* **[95% CI***^f^***]** | **NPV***^g^* **[95% CI]** |
| --- | --- | --- | --- | --- | --- | --- |
| All variants | 3688413 | 2571369697 | 3907 | 2448 | 99.89% [99.89% - 99.90%] | 100% [100% - 100%] |
| SNVs*^h^* only | 3207969 | 2571369697 | 2522 | 1346 | 99.92% [99.92% - 99.92%] | 100% [100% - 100%] |
| Insertions and deletions | 480444 | 2571369697 | 1385 | 1102 | 99.71% [99.70% - 99.73%] | 100% [100% - 100%] |
| Segmental duplications | 59054 | 43102836 | 375 | 329 | 99.37% [99.30% - 99.43%] | 100% [100% - 100%] |
| Low mappability regions | 172097 | 99469838 | 1458 | 1296 | 99.16% [99.12% - 99.20%] | 100% [100% - 100%] |
| Low complexity regions | 257660 | 62578838 | 879 | 823 | 99.66% [99.64% - 99.68%] | 100% [100% - 100%] |
| Low GC regions | 179834 | 120814998 | 276 | 211 | 99.85% [99.83% - 99.86%] | 100% [100% - 100%] |
| High GC regions | 413 | 377579 | 5 | 0 | 98.80% [97.23% - 99.61%] | 100% [100% - 100%] |
| Heterozygous variants only | 2233008 | 2571369697 | 3614 | 1621 | 99.84% [99.83% - 99.84%] | 100% [100% - 100%] |
| Homozygous variants only | 1454259 | 2571369697 | 256 | 790 | 99.98% [99.98% - 99.98%] | 100% [100% - 100%] |

*^a^*TP, true positive. *^b^*TN, true negative. *^c^*FP, false positive. *^d^*FN, false negative. *^e^*PPV, positive predictive value. *^f^*CI, confidence interval. *^g^*NPV, negative predictive agreement. *^g^*SNVs, single-nucleotide variants.

Table S22. Performance of different extraction platforms and input material types

| **Category** | **Type** | **Panel+***^a^***/WGS–***^b^* | **Panel– /WGS+** | **Panel+ /WGS+** | **Panel– /WGS–** | **PPA***^c^* **[95% CI***^d^***]** | **NPA***^e^* **[95% CI]** |
| --- | --- | --- | --- | --- | --- | --- | --- |
| Autogen | SNV*^g^* | 11 | 29 | 4340 | 3138045 | 99.8% [99.6%-99.9%] | 100% [100%-100%] |
| Chemagen | SNV | 9 | 11 | 3489 | 3138916 | 99.8% [99.6%-99.9%] | 100% [100%-100%] |
| Autogen | InDel*^h^* | 3 | 33 | 114 | 3142275 | 98.1% [96.1%-100%] | 100% [100%-100%] |
| Chemagen | InDel | 0 | 26 | 72 | 3142327 | 100% [100%-100%] | 100% [100%-100%] |
| WBC*^f^* | SNV | 11 | 11 | 3816 | 3138587 | 99.7% [99.5%-99.8%] | 100% [100%-100%] |
| Whole Blood | SNV | 12 | 29 | 4013 | 3138371 | 99.7% [99.5%-99.9%] | 100% [100%-100%] |
| WBC | InDel | 3 | 29 | 90 | 3142303 | 98.5% [96.8%-100%] | 100% [100%-100%] |
| Whole Blood | InDel | 0 | 30 | 96 | 3142299 | 100% [100%-100%] | 100% [100%-100%] |

*^a^+* indicates that a variant was present on the panel or the corresponding whole genome sequencing (WGS) sample. *^b^*– indicates that a variant was not present on the panel or the corresponding WGS sample. *^c^*PPA, positive percent agreement. *^d^*CI, confidence interval. *^e^*NPA, negative percent agreement. *^f^*WBC, whole blood and buffy coat. *^g^*SNVs, single nucleotide variants. *^h^*InDel, insertion or deletion.

Table S23. Mismatched bases after liftover between reference builds

|  | **GRCh37** | | | | **GRCh38** | | | |
| --- | --- | --- | --- | --- | --- | --- | --- | --- |
| **Gene** | **Chr** | **Start** | **End** | **Sequence** | **Chr** | **Start** | **End** | **Sequence** |
| *APOB* | chr2 | 21,235,474 | 21,235,475 | T | chr2 | 21,012,602 | 21,012,603 | C |
| *DSP* | chr6 | 7,563,982 | 7,563,983 | T | chr6 | 7,563,749 | 7,563,750 | G |
| *FBN1* | chr15 | 48,807,636 | 48,807,637 | C | chr15 | 48,515,439 | 48,515,440 | T |
| *TNNI3* | chr19 | 55,665,583 | 55,665,584 | A | chr19 | 55,154,215 | 55,154,216 | C |

Table S24. Change management process, by risk categorization

| **Category** | **Possible types of changes** | **Definition / Examples** | **Change level** | **Type of FDA notification** |
| --- | --- | --- | --- | --- |
| **Enrollment** | Significant clinical protocol change | Change in intended use / indication, reduction in sample size, change in method of estimation, early termination of study | Major | Supplement |
|  | Consent form content | Change of consent as new risk of results is identified, collection procedure | Moderate | 5-day notice |
| **Specimen collection** | Specimen collection kits | New brand of collection kits (if switching from a validated kit to a different product validated for the same use) | Minor | Annual |
| **Specimen quality** | New acceptable specimen type | Adding saliva or buccal swabs | Major | Supplement |
|  | Specimen intake rejection criteria | The Biobank adding a policy to reject frozen blood samples | Major | Supplement |
|  | Specimen intake procedures | The Biobank changing shipping instructions, courier | Minor | Annual |
| **DNA quality and quantity** | Acceptance criteria | Adjust the cut-off of A260/280, quantity or quality of DNA | Major | Supplement |
|  | Quantification method | Using a new method other than Picogreen | Minor | Annual |
| **Instruments and methods** | Sequencer change | Using sequencing other than Novaseq | Major | Supplement |
|  | Instrument script/program changes | Scripts change to optimize liquid handling | Minor | Annual |
| **Pipeline** | Components of pipeline | Changing the variant caller, aligner, reference genome | Moderate | 5-day notice |
|  | Reportable variant types | Adding structural or copy number variants | Major | Supplement |
|  | Software version updates | Bioinformatic pipeline updates | Minor | Annual |
| **Report** | Cosmetic format changes | *All of Us* logo revisions, moving sections within the report, adding a section which does not include clinical information | Minor | Annual |
|  | Return of results process | The Genome Centers return reports directly to participants | Major | Supplement |

Table S25. Lessons learned during the pre-submission process

| **Initial expectation** | **Components of IDE application** |
| --- | --- |
| Clearly identifying results as part of a research study mitigates clinical risk. | The research nature of the program does mitigate risk, but throughout the IDE process, we had to make significant specific changes to the text to emphasize this point. |
| Pre-analytical (e.g. biobanking) and post-analytical steps (e.g., confirmatory testing, report generation) are not a part of a WGS*^a^* ‘medical device’. | The entire genomic workflow from sample receipt to return of results was included. |
| The program would be composed of multiple investigational devices. | The program would be composed of a single investigational device. To that end, the Genome Centers performed several studies to demonstrate precision across sites. |
| Blood and saliva samples would be included in the submission. | Only blood samples were included as additional studies were required for saliva as a sample type. |
| Control samples could be used to determine sensitivity, specificity, positive predictive value, repeatability, reproducibility ,and limit of detection. | Validation criteria must be broken down by variant type and genomic context. |
| Samples used in validation studies should provide coverage of typical reporting results. | Validation cohort must reflect the variant prevalence of the population being tested. 'Highly prevalent' variants should make up most of the validation set. The set of highly prevalent, clinically significant variants should be pre-specified. |
| Control samples from cell lines are acceptable. | Most data should come from samples of the same type that will be collected by the program. |
| Standards and guidelines from expert bodies relating to pharmacogenomics reporting could be used in the selection of reportable alleles. | Information conveyed to participants should be limited to drugs for which there is information in the FDA*^b^* approved drug labeling that describes how genetic information can be used. |
| Standard clinical genetic testing reporting practices are acceptable. | The program must demonstrate that participants understand the reports and perform comprehension testing in a genetic testing-naive cohort that reflects the diversity of the program’s participants. |
| Future validations out of scope of the IDE.*^c^* | An explanation of how reporting will change in the future was included. |
| Existing quality metrics used for clinical laboratories were acceptable. | For quality metrics, a data-driven explanation of the specific cutoff selection was required. |
| There is a set standard to meet, as determined by the FDA. | With no predicate test, developing the standard to meet was a highly iterative process in collaboration with the FDA. |

*^a^*WGS, whole genome sequencing. *^b^*FDA, United States Food and Drug Administration. *^c^*IDE, investigational device exemption.

## Supplemental Figures and Figure Legends

[Figure S1. Number of true positive variants observed in the Hereditary Disease Risk genes.](https://drive.google.com/file/d/1IYnrN_uQpnTi_MuMZzrMaqshLQW_zWPs/view?usp=sharing)


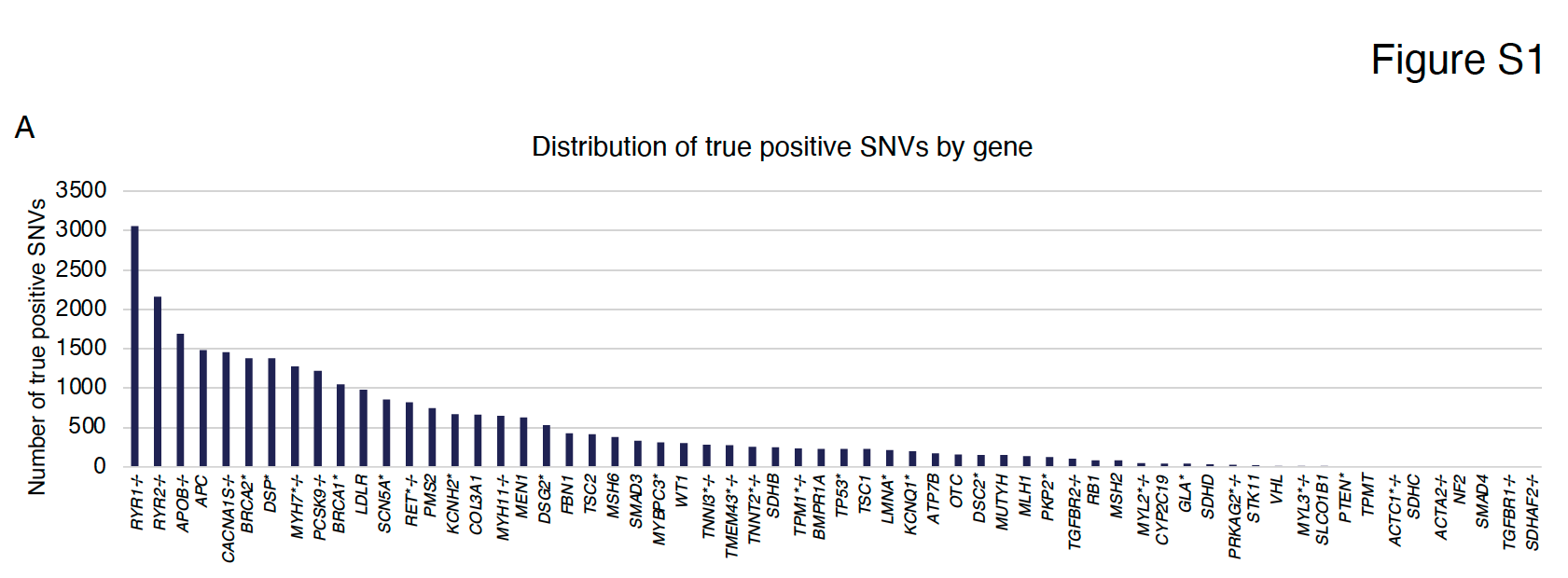


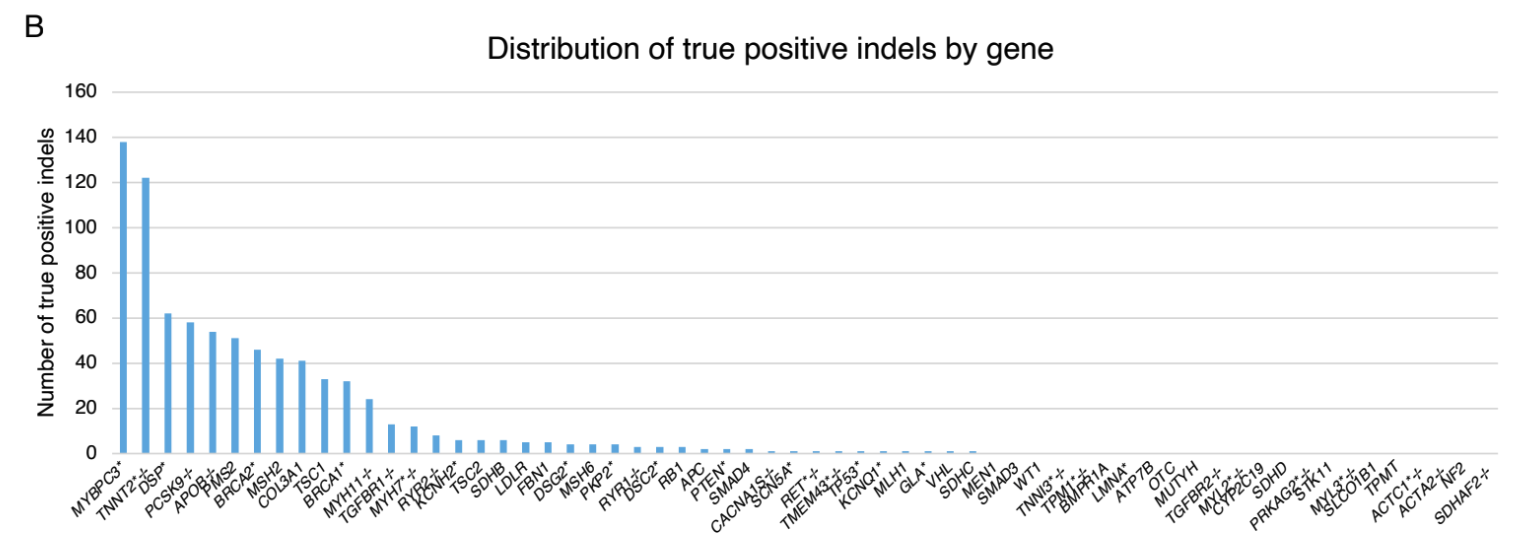


Data from a previous study was used to assess the likely frequency of reportable variants in the HDR genes. At least one true positive A) single nucleotide variant (SNV) or B) insertion or deletion (indel) was observed in each of the Hereditary Disease Risk (HDR) genes. Three additional genes not in the HDR gene list (*SLCO1B1, TMPT,* and *CYP2C19*) were included as they were on the orthogonal gene panels used for comparison. * indicates genes with potential surgical interventions indicated for a pathogenic/likely pathogenic variant. ✢ indicates genes in which loss of function is not a known mechanism of disease pathogenesis, meaning insertions or deletions are typically not reportable.

[Figure S2. Performance of variant calling from WGS as a function of insertion size and deletion size.](https://drive.google.com/file/d/11uGcyIIh6-wjAF57yKdMEM_jhqaYD6Vf/view?usp=sharing)


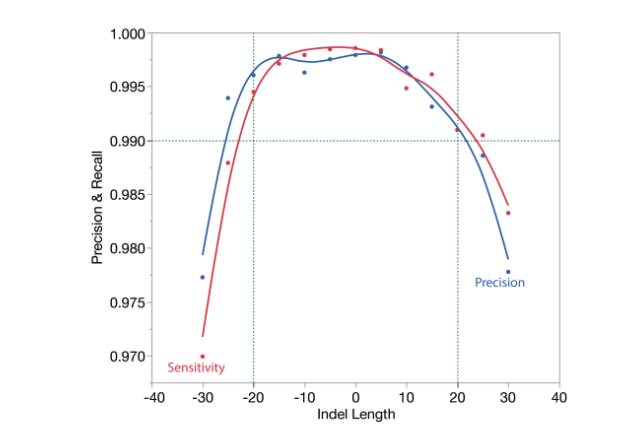


The accuracy of the AoURP device was >99% (horizontal dotted line) in events up to 20 bases in length (vertical dotted lines) and ≥97% in events out to 30 bases in length when compared to a well-established truth sample (NA12878). Red line indicates sensitivity, and blue line indicates precision.

[Figure S3. Sequence generation quality control specification determination.](https://drive.google.com/file/d/1yEKEn72JrpzDOJ1dbCeqKz1OAeFvdIVE/view?usp=sharing)


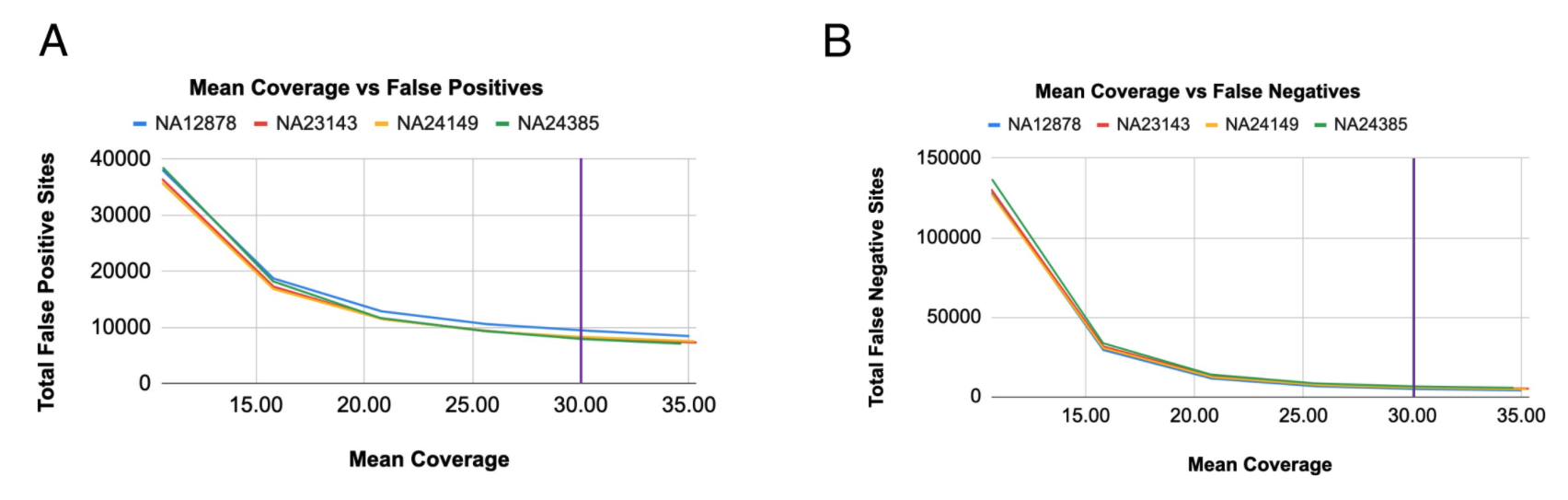


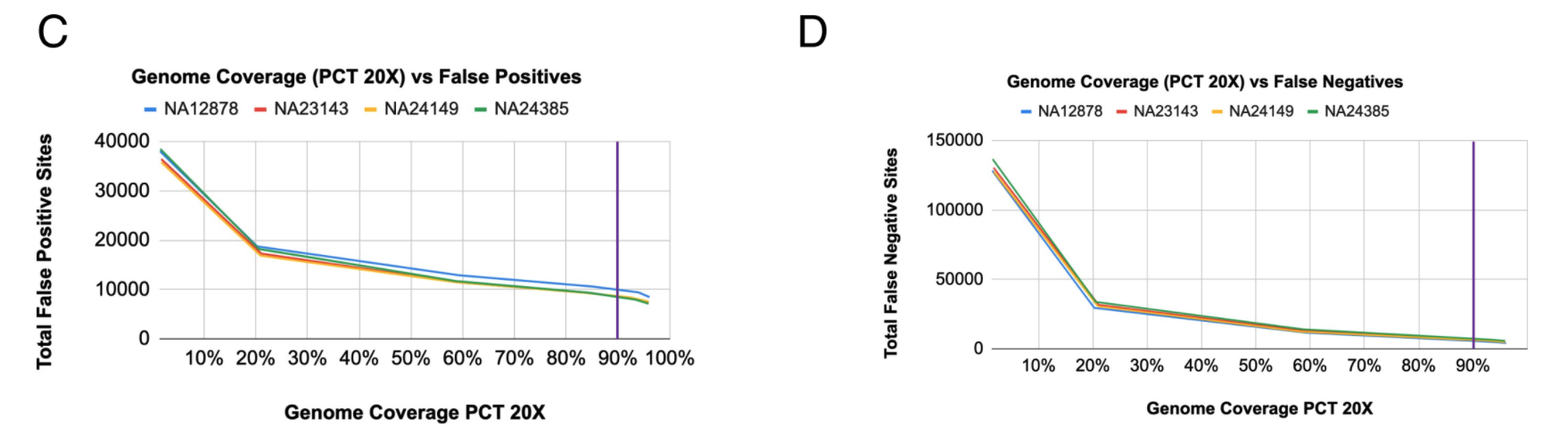


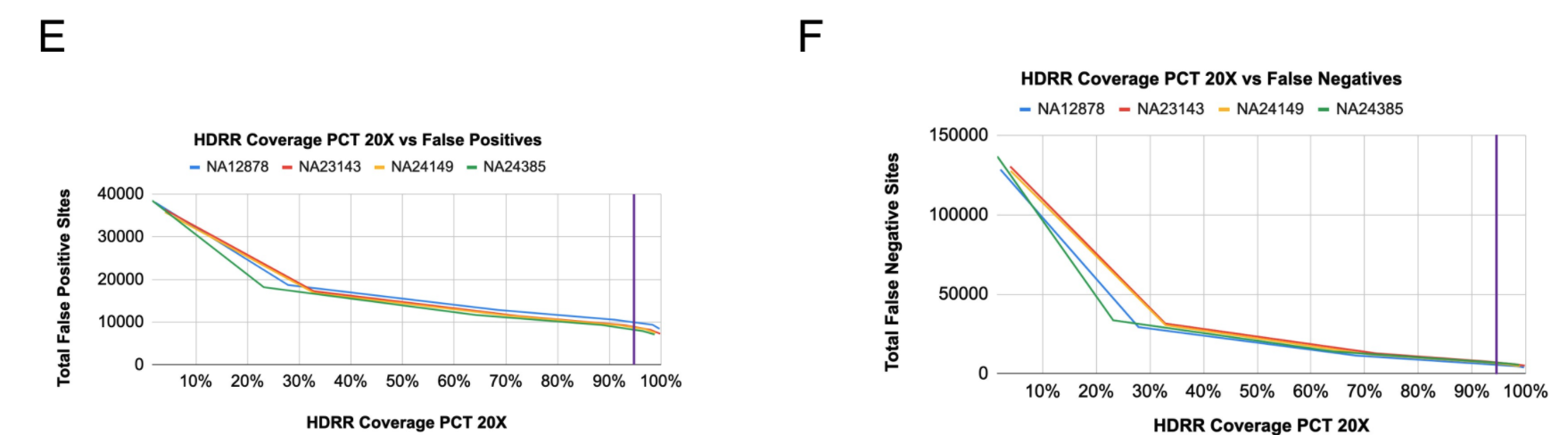


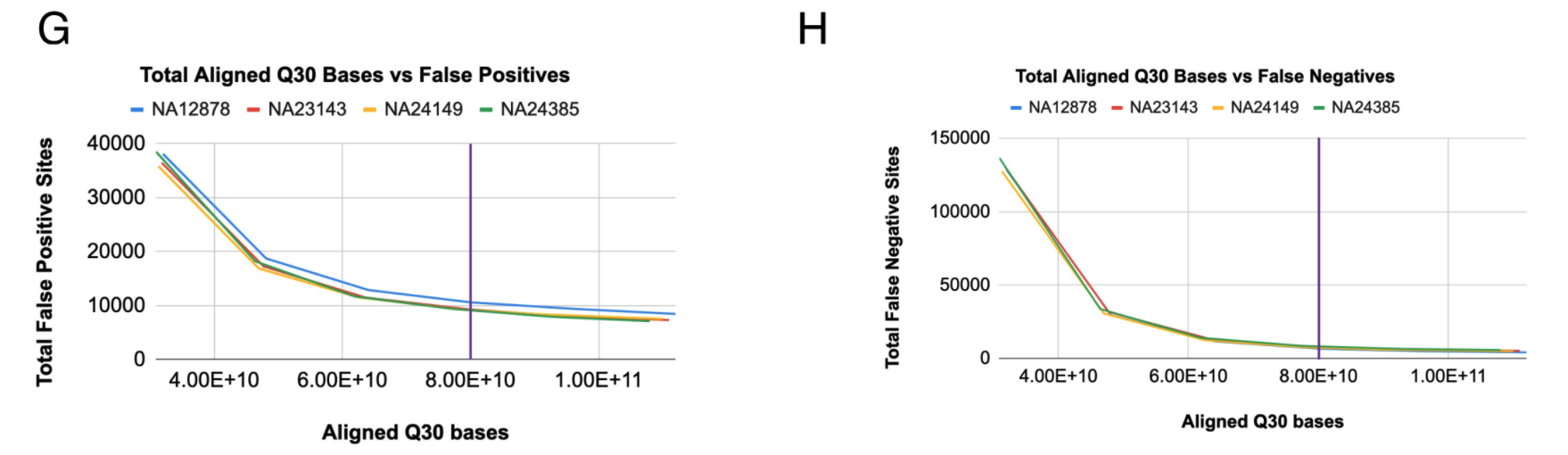


We generated data to demonstrate thresholds used for QC metrics. The total A) false positives and B) false negatives showed a gradual increase as mean coverage decreased, with a rapid increase below 20X coverage. The total C) false positives and D) false negatives steadily increased as the percentage of bases with at least 20X coverage decreased. The total E) false positives and F) false negatives increased gradually as the average coverage in Hereditary Disease Risk Report (HDRR) regions decreased. The reduction in performance was slow initially and then increased rapidly below 40%. The total G) false positives and H) false negatives increased with lower base quality counts, with inflection points starting around 6e10 for both. Vertical purple line marks the device acceptance criteria.

[Figure S4. Relationship between estimated sample contamination and performance.](https://drive.google.com/file/d/1bq473y6wKT6vpYvyeVKPzldWZ5kOyIAG/view?usp=sharing)


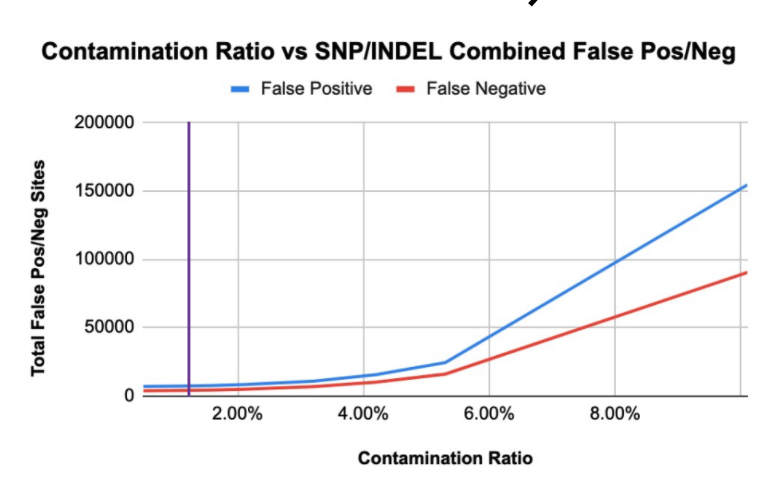


Variant calling performance, as measured by both the number of false positives (pos) and the number of false negatives (neg) for single nucleotide polymorphisms (SNPs) and insertions or deletions (indels), decreased with increasing contamination. Blue line indicates false positives, and red line indicates false negatives. Blue line indicates false positives, and red line indicates false negatives. Vertical purple line marks the device acceptance criteria.

[Figure S5. Relationship between duplicate rate and performance.](https://drive.google.com/file/d/1qkpMrlQplypJtv7YxCtrrrwfcsTPsdQH/view?usp=sharing)


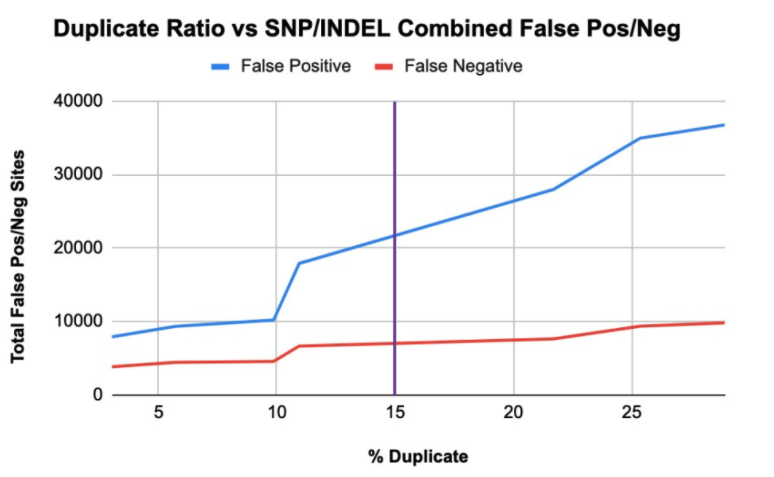


Samples with a higher percentage of duplicate reads had a higher total number of false positive (pos) and false negative (neg) single nucleotide polymorphisms (SNPs) and insertions or deletions (indels). Blue line indicates false positives, and red line indicates false negatives. Vertical purple line marks the device acceptance criteria.

[Figure S6. Relationship between mean coverage and uniformity.](https://drive.google.com/file/d/1Ps9TcF4erNDNJoZi7JlMA761V4ScDvp1/view?usp=sharing)


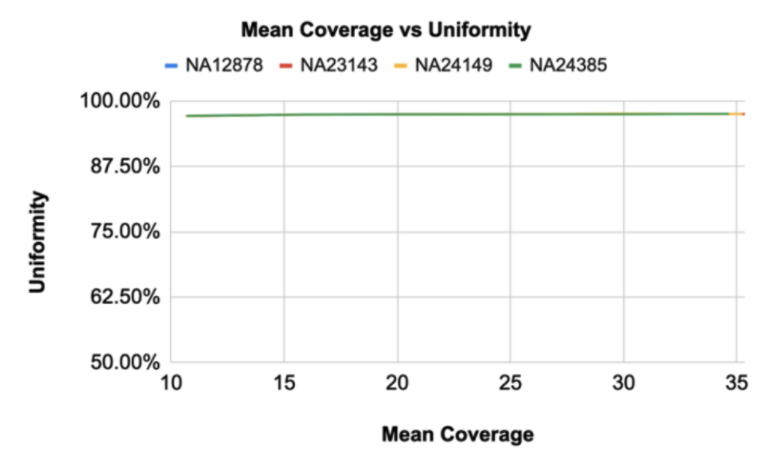


The uniformity metric did not deviate as data quality was reduced in four well-established truth samples (NA12878, NA23143, NA24149, and NA24385).

[Figure S7. Analytical sensitivity for NA12878 input titration series vs NIST across all three Genome Centers.](https://drive.google.com/file/d/1JIFyNCQ0WtPnpYWmPmhUIS2ZVaBX_Fd4/view?usp=sharing)


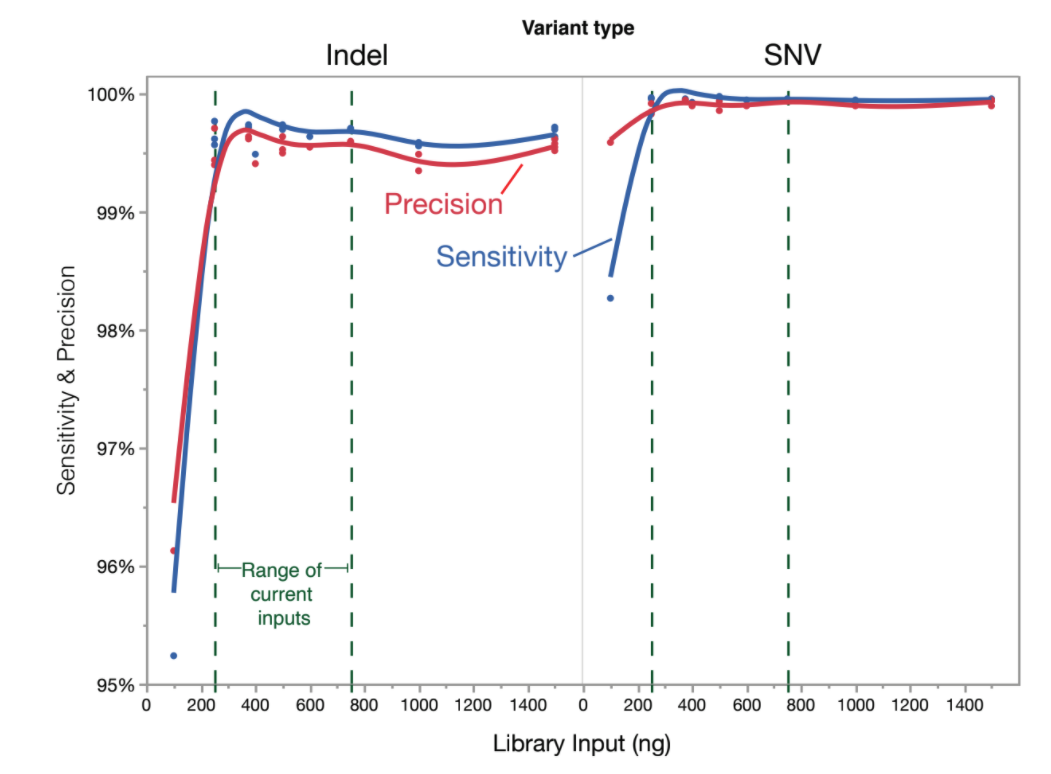


Library input amounts below 250 ng showed reduced sensitivity. There was no significant difference in performance between 250 ng and 1500 ng input DNA. Red line indicates precision, and blue line indicates sensitivity. Indel, insertion or deletion. SNV, single nucleotide variant.

[Figure S8. Precision and recall (i.e., sensitivity) of SNV and indel calling as a function of alt allele fraction.](https://drive.google.com/file/d/1PFIiRTDulmZMfXAdXez0dl-9hF6mStba/view?usp=sharing)


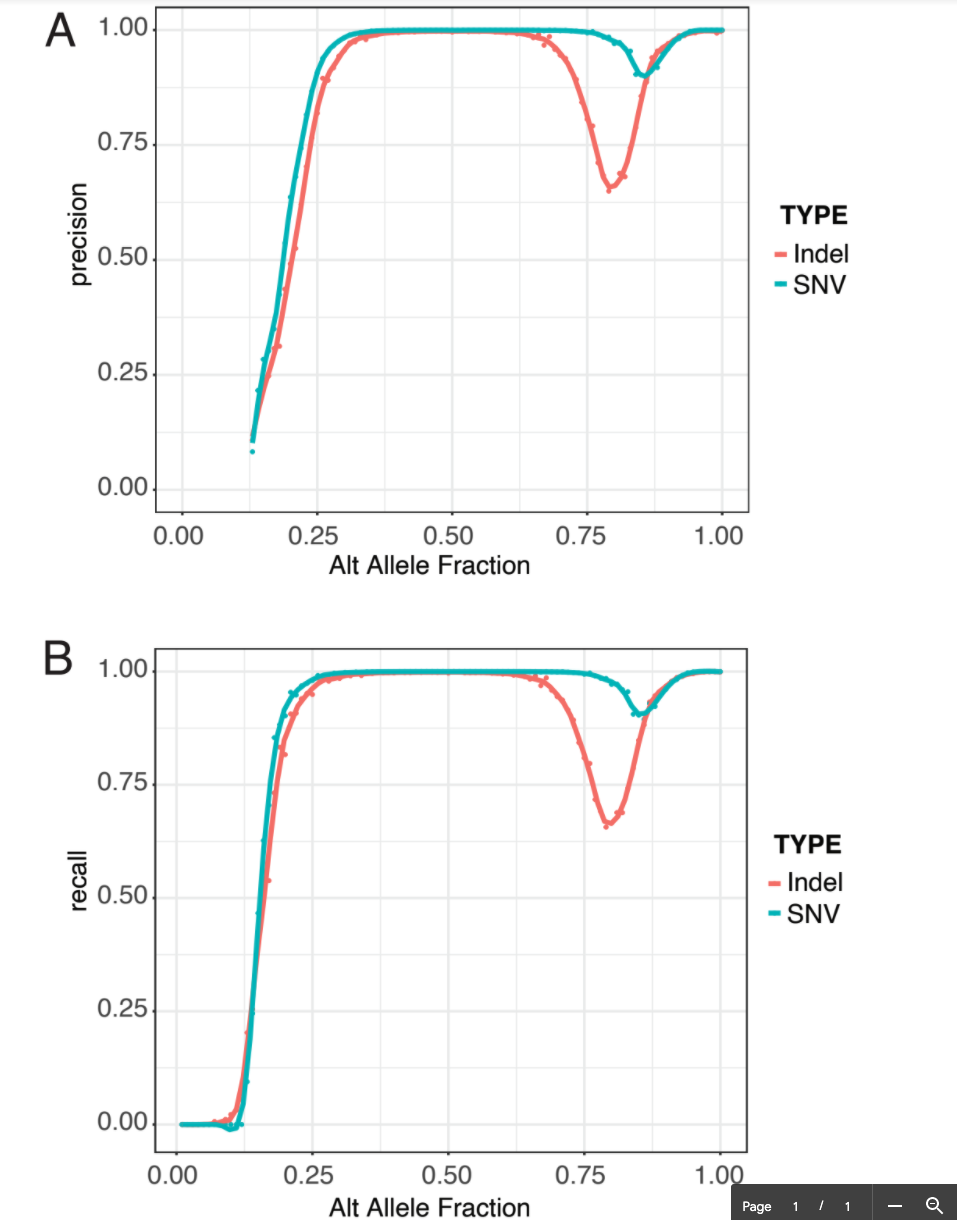


Heterozygous calls made between 30-75% allele fraction for single nucleotide variants (SNVs) and 30-65% allele fraction for insertions or deletions (indels) had high A) precision and B) recall. Peach line indicates indels, and teal line indicates SNPs. Alt, alternative.
